# Underexplored diversity of gill monogeneans in cichlids from Lake Tanganyika: eight new species of *Cichlidogyrus* Paperna, 1960 (Monogenea: Dactylogyridae) from the northern basin of the lake, with remarks on the vagina and the heel of the male copulatory organ

**DOI:** 10.1186/s13071-017-2460-6

**Published:** 2017-12-02

**Authors:** Chahrazed Rahmouni, Maarten P. M. Vanhove, Andrea Šimková

**Affiliations:** 1 Department of Botany and Zoology, Faculty of Science, Masaryk University, Kotlářská 2, CZ-611 37 Brno, Czech Republic; 2 Capacities for Biodiversity and Sustainable Development (CEBioS), Operational Directorate Natural Environment, Royal Belgian Institute of Natural Sciences, Vautierstraat 29, B-1000 Brussels, Belgium; 3 Laboratory of Biodiversity and Evolutionary Genomics, Department of Biology, University of Leuven, Charles Deberiotstraat 32, B-3000 Leuven, Belgium; 4 Centre for Environmental Sciences, Research Group Zoology: Biodiversity & Toxicology, Hasselt University, Agoralaan Gebouw D, B-3590 Diepenbeek, Belgium

**Keywords:** Africa, Burundi, Cichlidae, Cyprichromini, Ectodini, Eretmodini, Platyhelminthes, Monogenea, *Cichlidogyrus*

## Abstract

**Background:**

Lake Tanganyika harbours the most diverse cichlid assemblage of the Great African Lakes. Considering its cichlid flocks consist of approximately 250 endemic species, we can hypothesize a high species-richness in their often quite host-specific monogenean ectoparasites belonging to *Cichlidogyrus* Paperna, 1960. Yet, only 24 species were described from Tanganyikan hosts and some host tribes have never been investigated for monogeneans. This study presents the first parasitological examination of species of the tribes Cyprichromini (*Cyprichromis microlepidotus* (Poll, 1956)), Eretmodini (*Eretmodus marksmithi* Burgess, 2012 and *Tanganicodus irsacae* Poll, 1950) and Ectodini (*Aulonocranus dewindti* (Boulenger, 1899)). Specimens of the ectodine *Ophthalmotilapia nasuta* (Poll & Matthes, 1962) from which four *Cichlidogyrus* spp. have been previously described from more southern localities were also studied. Further, we discuss the haptor configuration in Tanganyikan *Cichlidogyrus* spp. and highlight the morphological diversity of the vagina, and that of the heel, a sclerotized part of the male copulatory organ, absent in some species of *Cichlidogyrus*.

**Methods:**

*Cichlidogyrus* spp. were isolated from gills and fixed using GAP. Haptoral and genital hard parts were measured and drawn by means of a phase contrast microscopic examination.

**Results:**

We describe eight new species: *Cichlidogyrus milangelnari* n. sp. on *C. microlepidotus*; *C. jeanloujustinei* n. sp. on *E. marksmithi*; *C. evikae* n. sp. on *T. irsacae*; *C. aspiralis* n. sp., *C. glacicremoratus* n. sp. and *C. rectangulus* n. sp. on *O. nasuta*; and *C. pseudoaspiralis* n. sp. and *C. discophonum* n. sp. on *A. dewindti*. Three haptoral morphotypes were recognized among the new species. Species of *Cichlidogyrus* from closely related hosts exhibited the same morphotypes. Geographical variation in *Cichlidogyrus* spp. fauna as observed in *O. nasuta* and three morphotypes were distinguished. Finally, we listed 111 *Cichlidogyrus* species, of which 27 and three Tanganyikan species lack sclerotized vagina and heel, respectively, just like 19 and seven species outside of the lake.

**Conclusions:**

Haptoral and genital features in the Tanganyikan *Cichlidogyrus* fauna reflect the phylogenetic relationships of their cichlid hosts. It seems that several lineages of *Cichlidogyrus* spp. exist in Lake Tanganyika but further studies are necessary to confirm this hypothesis and answer questions related to Lake Tanganyika and its cichlids.

**Electronic supplementary material:**

The online version of this article (10.1186/s13071-017-2460-6) contains supplementary material, which is available to authorized users.

## Background

The family Cichlidae Heckel, 1840 is one of the most species-rich families of vertebrates and is characterized by a high diversity in morphology, colours and behaviour [1]. With about 2200 described species (http://researcharchive.calacademy.org/research/ichthyology/catalog/SpeciesByFamily.asp) and their current disjunctive distribution from Central and South America, across Africa to Madagascar, the Middle East and the Indian subcontinent, they have attracted much attention of evolutionary biologists and ecologists [2, 3]. The East African Great Lakes Victoria, Malawi and Tanganyika, main hotspots of cichlid biodiversity, alone harbour more than 1500 endemic cichlid species and have therefore been the focus of numerous studies. Lake Tanganyika, the deepest and oldest lake in Africa, counts the genetically, morphologically and ecologically most diverse cichlid assemblages of these lakes [4]. With an estimated age of 9–12 million years (MY), it holds about 75 non-cichlid and 250 endemic cichlid fish species. The latter belong to more than 50 genera and 12 to 14 tribes [5, 6]. Cichlids have become one of the best models for the study of biological diversification and rapid radiation [2, 3, 7]. As host-parasite systems are suitable to furnish information on the evolution and distribution of the hosts as well as to elucidate the processes of parasite speciation, the parasites of cichlids are the objects of special scientific interest as well [8–10]. More than 100 African and Levantine cichlid species have been investigated for the presence of monogenean parasites [8, 11].

Overall, 13 monogenean genera were proposed from cichlid hosts worldwide. Six of them, i.e. *Urogyrus* Bilong Bilong, Birgi & Euzet, 1994; *Enterogyrus* Paperna, 1963; *Onchobdella* Paperna, 1968; *Scutogyrus* Pariselle & Euzet, 1995; *Cichlidogyrus* Paperna, 1960 (Dactylogyridae) and *Gyrodactylus* von Nordmann, 1832 (Gyrodactylidae), were recognized in African cichlids [3, 11]. Of these, *Cichlidogyrus* is the most species-rich with species infecting almost exclusively Cichlidae (a few *Cichlidogyrus* representatives occur also on Cyprinodontidae and Nandidae). More than 100 species of *Cichlidogyrus* were described from more than 100 cichlid hosts [11–13]. The parasite fauna of fishes in Lake Tanganyika is being systematically investigated since recently. To date, only a limited number of monogeneans was described, i.e. 24 *Cichlidogyrus* spp. from only 20 cichlid hosts [14]. At least six cichlid tribes were never investigated for monogeneans [15, 16]. The distribution of the gill monogeneans in Tanganyikan cichlids may provide additional evidence for the interrelationships among cichlid species [10, 17].

As for all monogenean parasites, the description of *Cichlidogyrus* spp. is mainly based on the morphology of the sclerotized structures of the attachment organ (i.e. haptor) and reproductive organs (i.e. vagina and male copulatory organ, MCO) [18]. The haptoral structures in *Cichlidogyrus* spp. seem to be characteristic for major phylogenetic lineages, while the MCO is important for species-level identification [12, 17, 19]. In addition, these haptoral structures in dactylogyridean monogeneans have been extensively studied in various ecological and evolutionary contexts because of their influence on the host specificity, parasite specialization and reproductive isolation among congeners through niche ecology [12, 20–22]. Haptoral structures and MCO in *Cichlidogyrus* spp. present a high morphological diversity in terms of shape and size. The haptor of an adult specimen comprises two pairs of anchors (also termed gripi) (one dorsal and one ventral), two transversal bars (dorsal bar with two typical auricles and a V-shaped ventral bar) and seven dorsal and ventral pairs of hooks (also termed uncinuli). Four main morphological groups were recognized by Vignon et al. [21] based on the configuration of the hook pairs. The vagina in *Cichlidogyrus* spp. can be sclerotized or not [11]. The MCO consists of two main parts, i.e. copulatory tube and accessory piece (not always present; see [23]). The copulatory tube has an ovoid basal bulb in the proximal part prolonged into a tube of variable size and shape, with a simple or ornamented distal end. The accessory piece normally extends from the basal bulb and presents a simple or complicated structure [24–27]. The sclerotized portion basal to the ovoid bulb, commonly called “heel”, is relevant to species identification in *Cichlidogyrus*. This structure, because of sclerotization, was often (but not always), considered as associated to the accessory piece. The presence of this sclerotized portion as a part of the MCO was reported in the original descriptions of *Cichlidogyrus* spp. from various cichlid hosts (e.g. [23, 25–31]). All species of *Cichlidogyrus* described until now from Tanganyikan cichlid hosts possess a heel (e.g. [14, 28]) except for *Cichlidogyrus attenboroughi* Kmentová, Gelnar, Koblmüller & Vanhove, 2016 from *Benthochromis horii* Takahashi, 2008 (Benthochromini) which was described recently [16].

The aim of the present research was to study the gill monogeneans belonging to *Cichlidogyrus* spp. in littoral cichlid fish communities of Lake Tanganyika in Burundi. Only scarce reports on these flatworms exist from this stretch of the lakeshore [10, 13, 16]. Investigation of five Tanganyikan hosts of three different tribes, i.e. *Cyprichromis microlepidotus* (Poll, 1956) (Cyprichromini), *Eretmodus marksmithi* Burgess, 2012 and *Tanganicodus irsacae* Poll, 1950 (Eretmodini), and *Aulonocranus dewindti* (Boulenger, 1899) and *Ophthalmotilapia nasuta* (Poll & Matthes, 1962) (Ectodini), allowed to record eight unknown *Cichlidogyrus* spp. which are described here. These hosts, except for *O. nasuta*, were investigated for parasites for the first time. Indeed, three species of *Ophthalmotilapia* Pellegrin, 1904 have already been investigated and four *Cichlidogyrus* species were previously described by Vanhove et al. [23] along the coasts of Lake Tanganyika in Congo, Zambia and Tanzania. Therefore, the present paper provides additional data on the high *Cichlidogyrus* spp. richness of Tanganyikan cichlids, and on geographical variation in parasite fauna throughout the lake. Finally, we discussed the configuration of the hook pairs (size and form, see [21]) in the newly described species and the importance of the morphological diversity of the vagina and heel for *Cichlidogyrus* systematics by indexing all species described so far as well as their type-hosts, type-localities, and reporting the characterization of their vagina and heel structures, based on the original descriptions and/or drawings.

## Methods

**Fig. 1:**
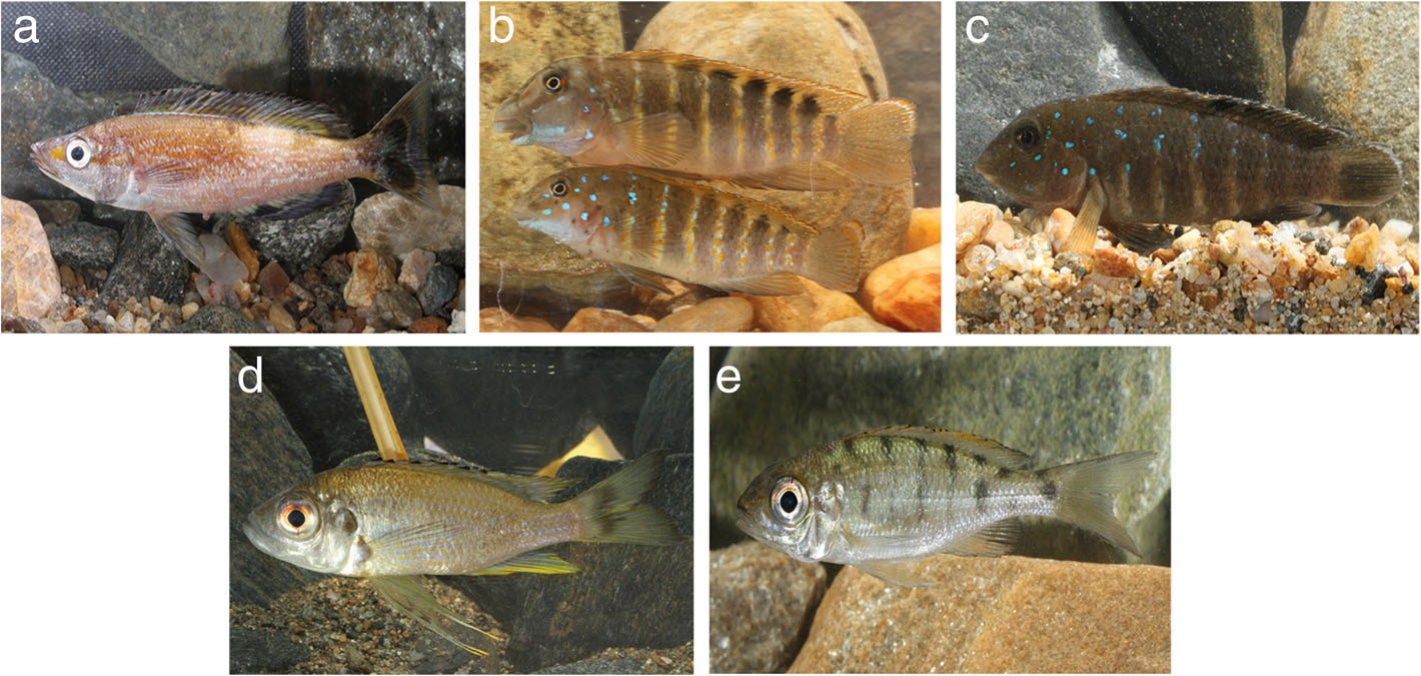
Cichlid hosts examined for the present study. **a** *Cyprichromis microlepidotus* (Poll, 1956). **b** *Eretmodus marksmithi* Burgess, 2012. **c** *Tanganicodus irsacae* Poll, 1950. **d** *Ophthalmotilapia nasuta* (Poll & Matthes, 1962)*.* **e** *Aulonocranus dewindti* (Boulenger, 1899). Photos by R. Blažek (Burundi, 2013)

**Fig. 2:**
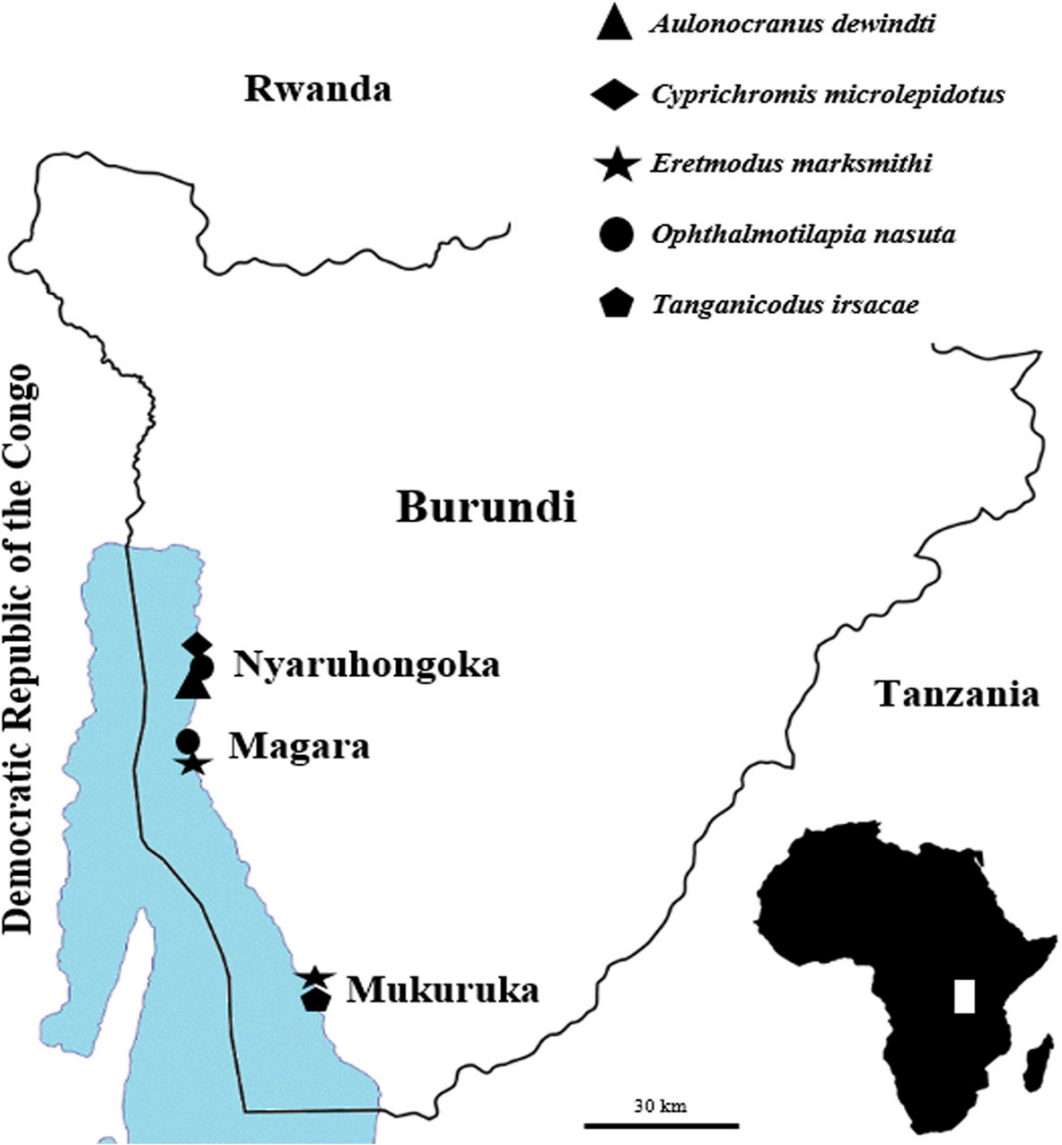
Map of Lake Tanganyika (*blue*) indicating the localities of sampling along the coast in Burundi

**Fig. 3:**
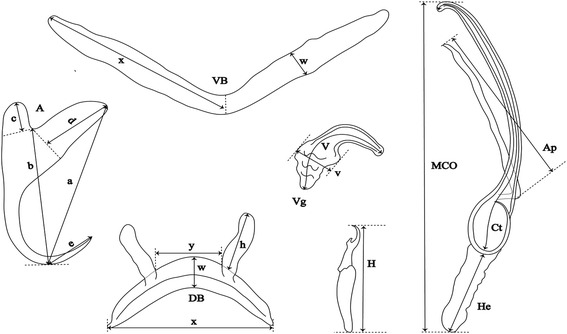
Measurements used in the descriptions of the new species of *Cichlidogyrus*. *Abbreviations*: A, anchor (DA, dorsal anchor; VA, ventral anchor; a, total length; b, blade length; c, shaft length; d, guard length; e, point length); DB, dorsal bar (h, auricle length; w; maximum straight width; x, total length; y, distance between auricles); VB, ventral bar (x, length of one ventral bar branch; w, maximum width); H, hook length; MCO, male copulatory organ straight length; Ct, copulatory tube curved length; He, heel straight length; Ap, accessory piece straight length; Vg, vagina (V, vagina total length; v, vagina width)

Cichlid specimens were acquired from commercial fishermen or caught using gill nets during snorkelling or diving in September 2013 in Burundi (Lake Tanganyika). When caught alive, they were sacrificed by severing the spinal cord. Cichlid hosts (Fig. 1) were identified *in situ* by Stephan Koblmüller (Karl-Franzens University of Graz, Austria) and photographs were taken by Radim Blažek (Institute of Vertebrate Biology, Czech Academy of Sciences, Czech Republic). Cichlid species investigated and their localities of sampling are detailed in Fig. 2. Gills were dissected by separating the gill arches via dorsal and ventral section using standard parasitological procedures, and transferred into a Petri dish containing water. Monogeneans were detached from the gills, isolated according to Musilová et al. [29] using an Olympus SZX7 stereomicroscope, mounted onto a slide according to Vanhove et al. [23] using a drop of glycerine ammonium picrate mixture (GAP) [30], and covered with a coverslip and sealed with nail polish. Measurements and photographs were taken at a magnification of ×1000 (objective ×100 oil immersion, ocular ×10), using an Olympus BX51 phase-contrast microscope and Olympus Stream Image Analysis v. 1.9.3 software. All measurements (included in the species descriptions) are presented in micrometres and given as the range followed by the mean and the number of specimens measured (*n*) in parentheses. Drawings of the haptoral sclerotized parts and the copulatory organ were made on flattened specimens using an Olympus BX51 microscope equipped with a drawing tube and edited with a graphic tablet compatible with Adobe Illustrator CS6 v. 16.0.0 and Adobe Photoshop v. 13.0. Terminology of haptoral sclerotized parts (i.e. anchors and hooks) follows Gussev [31]. The numbering of the hook pairs (Roman letters I-VII) is that recommended by Mizelle [32]. This method is preferred in adult specimens because it takes into consideration both antero-posterior and dorso-ventral positions of hooks [18, 33]. The length of the hook pairs i.e. “short” or “long” was assigned following Pariselle & Euzet [11]. The metrics used for the hard structures are shown in Fig. 3.

The type-material was deposited in the Invertebrate collection of the Royal Museum for Central Africa (RMCA), Tervuren, Belgium; the Muséum National d’Histoire Naturelle (MNHN), Paris, France; and the Iziko South African Museum (SAMC), Cape Town, Republic of South Africa. Prevalence and intensity of infection were calculated according to Bush et al. [34]. *Cichlidogyrus* species isolated from *Ophthalmotilapia* spp. in Burundi were compared to those previously described on representatives of *Ophthalmotilapia* spp. in Congo, Tanzania and Zambia by examination of museum specimens (Table 1). To highlight the importance of the vagina and heel in *Cichlidogyrus* species and give an overview of the morphological diversity of these sclerotized structures (shape and size when available), we looked for the reproductive organ features in the original descriptions and/or on drawings. These are provided in an alphabetic list of the African *Cichlidogyrus* species (from Tanganyika and elsewhere, see Additional file 1: Table S1), their type-hosts and type-localities, authors, and date of citation based mainly on the original descriptions and on the systematic revision of dactylogyridean cichlid parasites made by Pariselle & Euzet [11]. Host nomenclature follows FishBase [35].

**Table 1:** List of *Cichlidogyrus* spp. described from species of *Ophthalmotilapia* by Vanhove et al. [23]

*Cichlidogyrus* species	Host species	Locality	Material deposition
*Cichlidogyrus centesimus*	*Ophthalmotilapia ventralis*	Wonzye and Kasenga points (Zambia); Kikoti (D.R. Congo)	MRAC 37680 (paratype, 1 slide)
	*Ophthalmotilapia nasuta*; *O. boops*	Mtosi (Tanzania)	
*Cichlidogyrus makasai*	*O. ventralis*	Wonzye and Kasenga points (Zambia); Kikoti (D.R. Congo)	Paratype (2 slides) MRAC 37676 and 37,677
*Cichlidogyrus sturmbaueri*	*O. ventralis*	Wonzye and Kasenga points (Zambia)	Paratype (2 slides) MRAC 37681 and 37,682
	*O. nasuta*	Musamba (Tanzania)	
*Cichlidogyrus vandekerkhovei*	*O. ventralis*	Wonzye and Kasenga points (Zambia); Kikoti (D.R. Congo)	Paratype (2 slides) MRAC 37675 and 37,679
	*O. nasuta*; *O. boops*	Mtosi (Tanzania)	

## Results

Investigation of the five cichlid host species revealed the presence of eight new species of *Cichlidogyrus*: *C. milangelnari* n. sp. on *Cyprichromis microlepidotus* (Cyprichromini); *C. jeanloujustinei* n. sp. on *Eretmodus marksmithi* (Eretmodini); *C. evikae* n. sp. on *Tanganicodus irsacae* (Eretmodini); *C. aspiralis* n. sp., *C. glacicremoratus* n. sp. and *C. rectangulus* n. sp. on *Ophthalmotilapia nasuta* (Ectodini); and *C. pseudoaspiralis* n. sp. and *C. discophonum* n. sp. on *Aulonocranus dewindti* (Ectodini).

**Family Dactylogyridae Bychowski, 1933**

**Genus** ***Cichlidogyrus*** **Paperna, 1960**

### *Cichlidogyrus milangelnari* n. sp.

- ***Type-host*** **:** *Cyprichromis microlepidotus* (Poll, 1956) (Fig. 1a); tribe Cyprichromini (Perciformes: Cichlidae).
- ***Type-locality*** **:** Nyaruhongoka (3°41′S, 29°20′E), Lake Tanganyika, Burundi.
- ***Type-material*** **:** Holotype: MRAC_vermes_37940. Paratypes: MRAC_vermes_37940; MNHN HEL583; SAMC-A088695.
- ***Site in host*** **:** Gills.
- ***Prevalence and intensity of infection*** **:** 100% (3/3); 7–37 monogeneans per infected host.
- ***ZooBank registration*** **:** To comply with the regulations set out in article 8.5 of the amended 2012 version of the *International Code of Zoological Nomenclature* (ICZN) [36], details of the new species have been submitted to ZooBank. The Life Science Identifier (LSID) of the article is urn:lsid:zoobank.org:pub:3B9F16F6-8E3F-44F5-8D5D-B1D4A4754242. The LSID for the new name *Cichlidogyrus milangelnari* is urn:lsid:zoobank.org:act:789E0B84-18E1-46A4-8029-FC7A63140721.
- ***Etymology*** **:** The specific epithet of the new species, “*milangelnari*”, honors the Czech parasitologist Professor Milan Gelnar, head of the Laboratory of Parasitology (Department of Botany and Zoology, Faculty of Science, Masaryk University, Czech Republic) as the recognition for his kind guidance and daily support for the research on monogeneans in Lake Tanganyika.

### Description

**Fig. 4:**
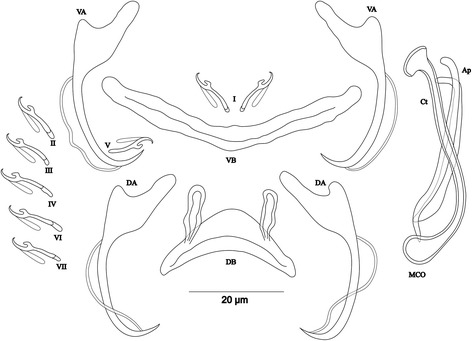
Sclerotized structures of *Cichlidogyrus milangelnari* n. sp. ex *Cyprichromis microlepidotus*. *Abbreviations*: DA, dorsal anchor; DB, dorsal bar; VA, ventral anchor; VB, ventral bar; I-VII, hooks; MCO, male copulatory organ; Ct, copulatory tube; Ap, accessory piece

[Based on 13 specimens fixed in GAP; Fig. 4]. Body 412–826 (597; *n* = 10) long, 69–156 (98; *n* = 10) wide at mid-body. Dorsal anchors with short shaft and more pronounced guard (*c.*2 times length of shaft) and curved blade with arched point: a = 33–36 (35; *n* = 12); b = 19–30 (23; *n* = 12); c = 2–6 (4; *n* = 11); d = 9–13 (10; *n* = 11); e = 5–7 (6; *n* = 12). Dorsal bar relatively small, curved, thick in middle part, with short auricles: h = 6–9 (9; *n* = 12); w = 7–9 (8; *n* = 12); x = 26–31 (29; *n* = 12); y = 11–14 (12; *n* = 12). Ventral anchors similar to dorsal ones: a = 32–35 (33; *n* = 12); b = 22–33 (29; *n* = 12); c = 1–6 (4; *n* = 12), d = 5–10 (8; *n* = 12); e = 6–9 (8; *n* = 12). Ventral bar V-shaped, with constant width: w = 4–9 (5; *n* = 12); x = 26–31 (29; *n* = 12). Haptor with 7 pairs of short hooks, hooks V with larval size (sensu [11, 37]); each hook with erect thumb and shank comprised of 2 subunits: pair I = 8–12 (10; *n* = 12) long, pair II = 10–12 (11; *n* = 12) long, pair III = 9–12 (11; *n* = 12) long, pair IV = 11–13 (12; *n* = 12) long, pair V = 7–11 (9; *n* = 12) long, pair VI = 10–13 (12; *n* = 12) long, pair VII = 9–12 (11; *n* = 12) long. Male copulatory organ composed of long copulatory tube with thick wall, associated to small bulb, curved at proximal third with fan-like ending: MCO = 39–45 (41; *n* = 13); Ct = 44–52 (48; *n* = 13). Heel absent. Accessory piece thick, composed of 2 superimposed parts with forked ending, Ap = 34–38 (36; *n* = 13). Vagina non-sclerotized.

### Differential diagnosis

*Cichlidogyrus milangelnari* n. sp. belongs to the group of species with short hook pairs I-IV, VI and VII (sensu Vignon et al. [21]), a copulatory tube without a swollen proximal portion and non-sclerotized vagina (see [37]), just like *C. attenboroughi* Kmentová, Gelnar, Koblmüller & Vanhove, 2016 [16]; *C*. *banyankimbonai* Pariselle & Vanhove, 2015 [17]; *C. berminensis* Pariselle, Bitja Nyom & Bilong Bilong, 2013 [38]; *C*. *bifurcatus* Paperna, 1960 [24]; *C. brunnensis* Kmentová, Gelnar, Koblmüller & Vanhove, 2016 [16]; *C. buescheri* Pariselle & Vanhove, 2015 [15]; *C. consobrini* Jorissen, Pariselle & Vanhove, 2017 [39]; *C*. *fontanai* Pariselle & Euzet, 1997 [40]; *C. frankwillemsi* Pariselle & Vanhove, 2015 [17]; *C. franswittei* Pariselle & Vanhove, 2015 [17]; *C. georgesmertensi* Pariselle & Vanhove, 2015 [17]; *C*. *gillardinae* Muterezi Bukinga, Vanhove, Van Steenberge & Pariselle, 2012 [41]; *C*. *gistelincki* Gillardin, Vanhove, Pariselle, Huyse & Volckaert, 2011 [42]; *C. halli* Price & Kirk, 1967 [43]; *C*. *haplochromii* Paperna & Thurston, 1969 [44]; *C*. *irenae* Gillardin, Vanhove, Pariselle, Huyse & Volckaert, 2012 [42]; *C*. *longipenis* Paperna & Thurston, 1969 [44]; *C*. *makasai* Vanhove, Volckaert & Pariselle, 2011 [23]; *C*. *mulimbwai* Muterezi Bukinga, Vanhove, Van Steenberge & Pariselle, 2012 [41]; *C*. *muterezii* Pariselle & Vanhove, 2015 [17]; *C*. *nageus* Řehulková, Mendlová & Šimková, 2013 [18]; *C. raeymaekersi* Pariselle & Vanhove, 2015 [17]; *C*. *rognoni* Pariselle, Bilong Bilong & Euzet, 2003 [45]; C*. schreyenbrichardorum* Pariselle & Vanhove, 2015 [15]; *C. sanjeani* Pariselle & Euzet, 1997 [40]; *C. sigmocirrus* Pariselle, Bitja Nyom & Bilong Bilong, 2014 [46]; *C*. *steenbergei* Gillardin, Vanhove, Pariselle, Huyse & Volckaert, 2012 [42]; *C*. *tilapiae* Paperna, 1960 [24], *C*. *vandekerkhovei* Vanhove, Volckaert & Pariselle, 2011 [23]; and *C*. *vealli* Pariselle & Vanhove, 2015 [15]. *Cichlidogyrus attenboroughi* was the first record of *Cichlidogyrus* spp. from Lake Tanganyika lacking a heel [16]. However, the new species is easily distinguishable from the latter by the (i) length of the dorsal bar auricles (6–9 μm in *C. milangelnari* n. sp. *vs* 14–23 μm in *C. attenboroughi*), (ii) the MCO (curved at the proximal third with fan ending *vs* L-shaped, strongly curved halfway with constricted ending in *C. attenboroughi*), and (iii) the accessory piece (thick, composed of two superimposed parts with forked ending in *C. milangelnari* n. sp. *vs* C-shaped, broader than copulatory tube in *C. attenboroughi*).

### *Cichlidogyrus jeanloujustinei* n. sp.

- ***Type-host*** **:** *Eretmodus marksmithi* Burgess, 2012 (Fig. 1b); tribe Eretmodini (Perciformes: Cichlidae).
- ***Type-locality*** **:** Mukuruka (4°14′S, 29°33′E), Lake Tanganyika, Burundi.
- ***Type-material*** **:** Holotype: MRAC_vermes_37939. Paratypes MRAC_vermes_37947; MNHN HEL582; SAMC-A088694.
- ***Site in host*** **:** Gills.
- ***Prevalence and intensity of infection*** **:** 30% (11/36); 1–3 monogeneans per infected host.
- ***ZooBank registration*** **:** To comply with the regulations set out in article 8.5 of the amended 2012 version of the *International Code of Zoological Nomenclature* (ICZN) [36], details of the new species have been submitted to ZooBank. The Life Science Identifier (LSID) of the article is urn:lsid:zoobank.org:pub:3B9F16F6-8E3F-44F5-8D5D-B1D4A4754242. The LSID for the new name *Cichlidogyrus jeanloujustinei* is urn:lsid:zoobank.org:act:51509F53-0C47-48F5-9E26-4252B83565AE.
- ***Etymology*** **:** The specific epithet “*jeanloujustinei*” honors the French parasitologist Jean-Lou Justine, Professor at the Muséum National d’Histoire Naturelle, Paris, France, who is extensively studying the systematics and biodiversity of monogeneans, digeneans, and nematodes.

### Description

**Fig. 5:**
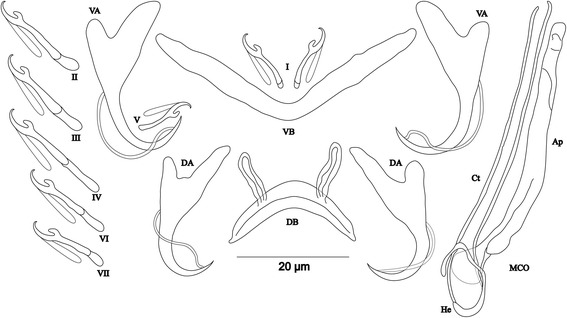
Sclerotized structures of *Cichlidogyrus jeanloujustinei* n. sp. ex *Eretmodus marksmithi*. *Abbreviations*: DA, dorsal anchor; DB, dorsal bar; VA, ventral anchor; VB, ventral bar; I-VII, hooks; MCO, male copulatory organ; He, heel; Ct, copulatory tube; Ap, accessory piece

[Based on 10 specimens fixed in GAP; Fig. 5]. Body 590–1397 (831; *n* = 5) long, 158–255 (194; *n* = 5) wide at mid-body. Dorsal anchors with short shaft and more pronounced guard (*c.*3 times length of shaft) and curved blade with arched point: a = 23–29 (25; *n* = 6); b = 16–20 (17; *n* = 6); c = 2–5 (4; *n* = 6), d = 8–13 (10; *n* = 6); e = 7–10 (8; *n* = 6). Dorsal bar relatively small, well arched, with short auricles: h = 7–9 (8; *n* = 6); w = 3–5 (4; *n* = 6); x = 21–25 (23; *n* = 6); y = 7–11 (9; *n* = 6). Ventral anchors with shaft shorter than guard, blade longer than in dorsal anchors, with arched point: a = 27–30 (28; *n* = 6); b = 21–25 (23; *n* = 6); c = 3–6 (4; *n* = 6); d = 8–11 (10; *n* = 6); e = 8–10 (9; *n* = 6). Ventral bar V-shaped, with constant width: w = 4–6 (5; *n* = 6); x = 27–35 (31; *n* = 6). Haptor with 7 pairs of short hooks, hooks V with larval size (see above); each hook with erect thumb and shank comprised of 2 subunits: pair I = 11–13 (12; *n* = 6) long, pair II = 15–19 (17; *n* = 6) long, pair III = 15–22 (19; *n* = 6) long, pair IV = 19–24 (21; *n* = 6) long, pair V = 10–12 (11; *n* = 6) long, pair VI = 18–23 (21; *n* = 6) long, pair VII = 15–18 (17; *n* = 6) long. Male copulatory organ composed of long, straight copulatory tube, associated to ovoid basal bulb with thick wall: MCO = 52–60 (56; *n* = 10); Ct = 51–59 (56; *n* = 10). Heel poorly developed, He = 1–2 (1; *n* = 9). Accessory piece proximally with 2 thick portions attached to basal bulb, slightly curved in middle distal part, with blunt ending, Ap = 41–45 (43; *n* = 10). Vagina non-sclerotized.

### Differential diagnosis

According to the relative length of the hook pairs, *C. jeanloujustinei* n. sp. belongs to the same morphological group as *C. milangelnari* n. sp. (see above). The characteristic structures of the MCO (reduced heel and two attached thick portions in the proximal part of the accessory piece) make *C. jeanloujustinei* n. sp. unique within this group. The new species exhibits haptoral structures similar to *C. milangelnari* n. sp. but differs in having shorter dorsal anchors (23–29 *vs* 33–36 μm) and a longer MCO (52–60 *vs* 39–45 μm).

### *Cichlidogyrus evikae* n. sp.

- ***Type-host*** **:** *Tanganicodus irsacae* Poll, 1950; tribe Eretmodini (Perciformes: Cichlidae) (Fig. 1c).
- ***Type-locality*** **:** Mukuruka (4°14′S, 29°33′E), Lake Tanganyika, Burundi.
- ***Type-material*** **:** Holotype: MRAC_vermes_37946. Paratypes: MRAC_vermes_37958; MNHN HEL586; SAMC-A088701.
- ***Site in host*** **:** Gills.
- ***Prevalence and intensity of infection*** **:** 71% (5/7); 1–3 monogeneans per infected host.
- ***ZooBank registration*** **:** To comply with the regulations set out in article 8.5 of the amended 2012 version of the *International Code of Zoological Nomenclature* (ICZN) [36], details of the new species have been submitted to ZooBank. The Life Science Identifier (LSID) of the article is urn:lsid:zoobank.org:pub:3B9F16F6-8E3F-44F5-8D5D-B1D4A4754242. The LSID for the new name *Cichlidogyrus evikae* is urn:lsid:zoobank.org:act:85036A92-0337-4428-9E88-6CC27E6205C2.
- ***Etymology*** **:** The name is given in honour of the Czech parasitologist Dr. Eva Řehulková (Department of Botany and Zoology, Faculty of Science, Masaryk University, Czech Republic) who studies monogenean flatworms for her contributions to our research.

### Description

**Fig. 6:**
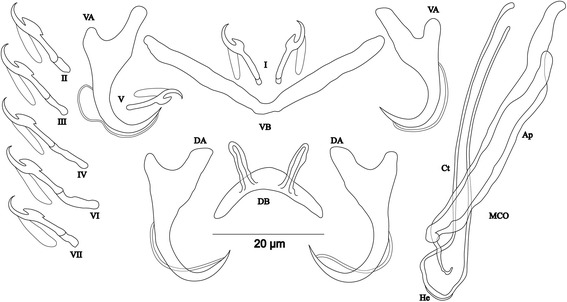
Sclerotized structures of *Cichlidogyrus evikae* n. sp. ex *Tanganicodus irsacae*. *Abbreviations*: DA, dorsal anchor; DB, dorsal bar; VA, ventral anchor; VB, ventral bar; I-VII, hooks; MCO, male copulatory organ; He, heel; Ct, copulatory tube; Ap, accessory piece

[Based on 12 specimens fixed in GAP; Fig. 6]. Body 400–1496 (883; *n* = 9) long, 109–305 (198; *n* = 9) wide at mid-body. Dorsal anchors with short shaft and more pronounced guard (*c.*2 times length of shaft) and curved blade with arched point: a = 20–23 (22; *n* = 10); b = 12–20 (16; *n* = 10); c = 3–5 (4; *n* = 10); d = 7–9 (8; *n* = 10); e = 7–9 (8; *n* = 10). Dorsal bar relatively small, well arched with equal thickness over entire width and short auricles: h = 6–10 (8; *n* = 10); w = 3–5 (4; *n* = 10); x = 17–26 (21; *n* = 10); y = 5–9 (7; *n* = 10). Ventral anchors with shaft shorter than guard, curved blade with arched point: a = 22–24 (23; *n* = 10); b = 19–21 (20; *n* = 10); c = 3–5 (4; *n* = 10); d = 6–9 (7; *n* = 10); e = 7–10 (8; *n* = 10). Ventral bar V-shaped: w = 3–6 (5; *n* = 10); x = 25–31 (28; *n* = 10). Haptor with 7 pairs of short hooks, hooks V with larval size (see above), thumb broad and junction with shank well pronounced with proximal protrusion: pair I = 11–13 (12; *n* = 11) long, pair II = 12–17 (15; *n* = 11) long, pair III = 15–19 (17; *n* = 11) long, pair IV = 19–23 (20; *n* = 6) long, pair V = 10–12 (11; *n* = 11) long, pair VI = 13–21 (18; *n* = 11) long, pair VII = 14–17 (16; *n* = 11) long. Male copulatory organ composed of long copulatory tube with thick wall, slightly curved where associated to irregularly shaped bulb, linked to accessory piece with thin filament: MCO = 53–58 (56; *n* = 12); Ct = 52–57 (54; *n* = 10). Heel reduced to inconspicuous, He = 0–2 (1; *n* = 12). Accessory piece with 2 distinct parts variable in thickness, superimposed with irregular surface, endings blunt, one extremity shorter than the other, Ap = 46–52 (49; *n* = 12). Vagina non-sclerotized.

### Differential diagnosis

*Cichlidogyrus evikae* n. sp. belongs to the same morphological group as *C. milangelnari* n. sp. and *C. jeanloujustinei* n. sp. (see above). It is most similar to *C. jeanloujustinei* n. sp. in having (i) a MCO with an ovoid basal bulb prolonged into a copulatory tube with thick wall, (ii) a poorly developed to inconspicuous heel, and (iii) an accessory piece composed of two superimposed parts. *Cichlidogyrus evikae* n. sp. can be distinguished from *C. jeanloujustinei* because the shape of the hook pairs (except for pairs I and V) is different: thumbs are broad in *C. evikae* n. sp. which gives an articulated appearance.

### *Cichlidogyrus aspiralis* n. sp.

- ***Type-host*** **:** *Ophthalmotilapia nasuta* (Poll & Matthes, 1962) (Fig. 1d); tribe Ectodini (Perciformes: Cichlidae).
- ***Type-locality*** **:** Magara (3°44′S, 29°19′E), Lake Tanganyika, Burundi.
- ***Type-material*** **:** Holotype: MRAC_vermes_37943; Paratype: MRAC_vermes_37954; SAMC-A088698.
- ***Site in host*** **:** Gills.
- ***Prevalence and intensity of infection*** **:** 75% (3/4); 1–2 monogeneans per infected host.
- ***ZooBank registration*** **:** To comply with the regulations set out in article 8.5 of the amended 2012 version of the *International Code of Zoological Nomenclature* (ICZN) [36], details of the new species have been submitted to ZooBank. The Life Science Identifier (LSID) of the article is urn:lsid:zoobank.org:pub:3B9F16F6-8E3F-44F5-8D5D-B1D4A4754242. The LSID for the new name *Cichlidogyrus aspiralis* is urn:lsid:zoobank.org:act:1918864B-1B58-4DCA-B97C-57A80C79A576.
- ***Etymology*** **:** The specific epithet “*aspiralis*” refers to the absence of a spiral thickening in the copulatory tube in comparison to the species *C. centesimus* described by Vanhove et al. [23].

### Description

**Fig. 7:**
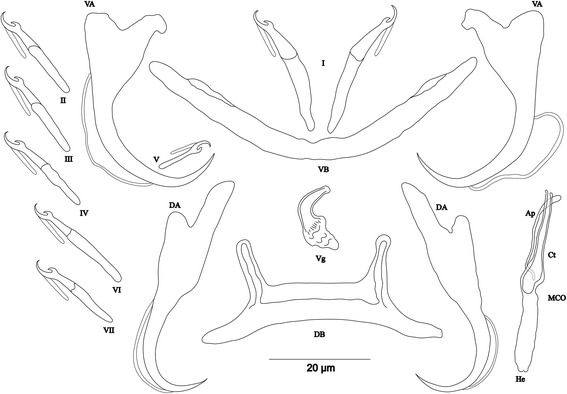
Sclerotized structures of *Cichlidogyrus aspiralis* n. sp. ex *Ophthalmotilapia asuta*. *Abbreviations*: DA, dorsal anchor; DB, dorsal bar; VA, ventral anchor; VB, ventral bar; I-VII, hooks; MCO, male copulatory organ; He, heel; Ct, copulatory tube; Ap, accessory piece; Vg, vagina

[Based on 4 specimens fixed in GAP; Fig. 7]. Body 336–407 (373; *n* = 3) long, 76–120 (102; *n* = 3) wide at mid-body. Dorsal anchors with short shaft and pronounced guard (*c.*4 times length of shaft) and blade slightly bent in middle with slightly curved point: a = 39–43 (41; *n* = 3); b = 32–33 (32; *n* = 3); c = 2–4 (3; *n* = 3); d = 10–14 (12; *n* = 3); e = 7–10 (9; *n* = 3). Dorsal bar long, straight with short appendages of anterior face of dorsal transverse bar: h = 6–10 (8; *n* = 3); w = 6–8 (7; *n* = 3); x = 45–47 (46; *n* = 3); y = 19–21 (20; *n* = 3). Ventral anchors with shorter shaft than guard and arched point: a = 33–34 (34; *n* = 3); b = 32–33 (33; *n* = 3); c = 2–5 (3; *n* = 3); d = 7–9 (8; *n* = 3); e = 9–10 (9; *n* = 3). Ventral bar V-shaped, with constant width: w = 4–5 (5; *n* = 3); x = 37–40 (39; *n* = 3). Hook pair I with well-developed shank, long in comparison with remaining pairs which are similarly short (sensu [11, 37]), pair V retains its larval size; each hook with erect thumb and shank comprised of 2 subunits: pair I = 26–27 (27; *n* = 3) long, pair II = 19–21 (20; *n* = 3) long, pair III = 20–23 (21; *n* = 3) long, pair IV = 20–21 (20; *n* = 3) long, pair V = 11–12 (12; *n* = 3) long, pair VI = 20–22 (21; *n* = 3) long, pair VII = 20–21 (20; *n* = 3) long. Male copulatory organ beginning in an ovoid bulb, with short straight copulatory tube: MCO = 36–38 (37; *n* = 4); Ct = 20–21 (21; *n* = 4). Heel long, straight, He = 14–18 (16; *n* = 4). Accessory piece thin, proximally connected to basal bulb, rounded and slightly enlarged distally, Ap = 14–19 (16; *n* = 4). Vagina short, sclerotized: V = 15–16 (15; *n* = 2); v = 5–7 (6; *n* = 2).

### Differential diagnosis

*Cichlidogyrus aspiralis* n. sp. belongs to the group of species exhibiting long hook pair I (pair V with larval size) and short pairs II-IV, VI and VII (see [21]), a copulatory tube without a swollen proximal portion, and a sclerotized vagina (see [37]). This group includes *C. albareti* Pariselle & Euzet, 1998 [47]; *C. dageti* Dossou & Birgi, 1984 [48]; *C. digitatus* Dossou, 1982 [49]; *C. dracolemma* Řehulková, Mendlová & Šimková, 2013 [18]; *C. euzeti* Dossou & Birgi, 1984 [48]; *C. falcifer* Dossou & Birgi, 1984 [48]; *C. longicirrus* Paperna, 1965 [50]; and *C. sanseoi* Pariselle & Euzet, 2004 [27]. The Tanganyikan species *C. muzumanii* isolated from *Tylochromis polylepis* (Boulenger, 1900) in the Congo is the only species hitherto known to have a long hook pair I and short pairs II-IV, VI and VII [41], and therefore *C. aspiralis* n. sp. is the second representative with this haptoral configuration in the Lake. In addition, the dorsal bar auricles in *C. aspiralis* n. sp. have small hollow outgrowths on the anterior face, a feature observed in congeners infecting representatives of *Tylochromis* Regan, 1920, such as the Tanganyikan *C. mulimbwai* and *C. muzumanii*, both parasites of *T. polylepis* [41] (see above), and the non-Tanganyikan *C. chrysopiformis*, *C. djietoi* and *C. sigmocirrus* Pariselle, Bitja Nyom & Bilong Bilong, 2014 from *T. sudanensis* Daget, 1945 [46], *C. kothiasi* Pariselle & Euzet, 1994 from *T. jentinki* (Steindachner, 1862) [51], and also *C. dageti*, C*. euzeti* and *C. falcifer* found on *Hemichromis fasciatus* Peters, 1857 [48]. *Cichlidogyrus aspiralis* n. sp. resembles *C. centesimus* Vanhove, Volckaert & Pariselle, 2011 isolated from *O. boops, O. nasuta* and *O. ventralis* [23] based on a similarly shaped MCO with a relatively slender and long heel. However, *C. aspiralis* n. sp. is mainly distinguishable from *C. centesimus* by (i) the absence of a spirally coiled thickening in the distal part of the copulatory tube (present in *C. centesimus*), and (ii) the presence of an accessory piece (absent in *C. centesimus*). *Cichlidogyrus aspiralis* n. sp. is also similar to *C. casuarinus* Pariselle, Muterezi Bukinga & Vanhove, 2015, described from *Bathybates minor* Boulenger, 1906, in having a similarly shaped dorsal bar (see above), a relatively long straight heel, and a sclerotized vagina. However, the new species is easily distinguishable from the latter by (i) the shorter dorsal (39–43 *vs* 52–64 μm) and ventral anchors (33–34 *vs* 47–59 μm, (ii) the shorter dorsal (45–47 *vs* 64–85 μm) and ventral bars (37–40 *vs* 54–67), (iii) the hook pair I (long and well-developed in *C. aspiralis* n. sp. *vs* long but not thick in *C. casuarinus*), (iv) the shorter heel (14–18 μm *vs* 40–59 μm), (v) the differently sized and shaped copulatory tube (short straight copulatory tube, 20–21 μm in *C. aspiralis* n. sp. *vs* straight and pointed, with distal external wall exhibiting a typical spirally coiled thickening, 34–44 μm in *C. casuarinus*), and (vi) the shorter and differently shaped accessory piece (thin, proximally connected to the basal bulb, rounded and slightly enlarged distally, 14–19 μm long in *C. aspiralis* n. sp. *vs* simple and thin often extending beyond penis and ending in a well developed, enlarged and bulbous extremity, attached by a filament to the distal extremity of the basal bulb, 26–38 μm long in *C. casuarinus*).

### *Cichlidogyrus glacicremoratus* n. sp.

- ***Type-host*** **:** *Ophthalmotilapia nasuta* (Poll & Matthes, 1962) (Fig. 1d); tribe Ectodini Perciformes: Cichlidae).
- ***Type-locality*** **:** Magara (3°44′S, 29°19′E), Lake Tanganyika, Burundi.
- ***Type-material*** **:** Holotype: MRAC_vermes_37941; Paratypes: MRAC_vermes_37942; MRAC_vermes_37948; MRAC_vermes_37950; MRAC_vermes_37952; MRAC_vermes_37953; MNHN HEL584; SAMC-A088696.
- ***Site in host*** **:** Gills.
- ***Prevalence and intensity of infection*** **:** 75% (3/4); 4–28 monogeneans per infected host.
- ***ZooBank registration*** **:** To comply with the regulations set out in article 8.5 of the amended 2012 version of the *International Code of Zoological Nomenclature* (ICZN) [36], details of the new species have been submitted to ZooBank. The Life Science Identifier (LSID) of the article is urn:lsid:zoobank.org:pub:3B9F16F6-8E3F-44F5-8D5D-B1D4A4754242. The LSID for the new name *Cichlidogyrus glacicremoratus* is urn:lsid:zoobank.org:act:54CA15E9-086B-4113-9C94-A56D47A14F51.
- ***Etymology*** **:** The specific epithet “*glacicremoratus*” is derived from the Latin “glacies” and “cremor” and refers to the shape of the proximal part of the MCO which reminds of an ice-cream.

### Description

**Fig. 8:**
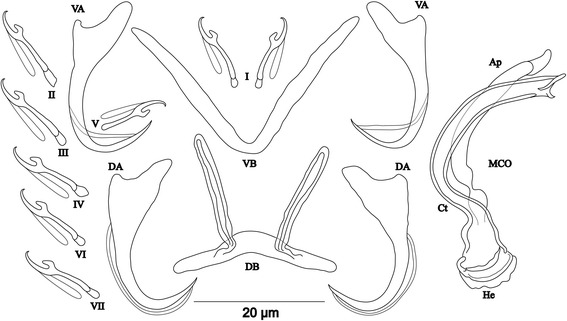
Sclerotized structures of *Cichlidogyrus glacicremoratus* n. sp. ex *Ophthalmotilapia nasuta*. *Abbreviations*: DA, dorsal anchor; DB, dorsal bar; VA, ventral anchor; VB, ventral bar; I-VII, hooks; MCO, male copulatory organ; He, heel; Ct, copulatory tube; Ap, accessory piece

[Based on 14 specimens fixed in GAP; Fig. 8]. Body 356–546 (459; *n* = 13) long, 88–145 (108; *n* = 13) wide at mid-body. Dorsal anchors relatively small with short shaft and more pronounced guard (*c.*2 times length of shaft), arched blade with slightly curved point: a = 19–23 (21; *n* = 13); b = 14–20 (18; *n* = 13); c = 1–3 (2; *n* = 13), d = 4–6 (5; *n* = 13); e = 7–10 (8; *n* = 13). Dorsal bar slightly curved, with straight long auricles: h = 14–18 (16; *n* = 13); w = 3–5 (4; *n* = 13); x = 23–27 (25; *n* = 13); y = 5–7 (6; *n* = 13). Ventral anchors with shaft shorter than guard and curved blade with arched point: a = 20–24 (21; *n* = 12); b = 17–19 (18; *n* = 12); c = 1–3 (2; *n* = 12), d = 4–6 (5; *n* = 12); e = 7–9 (8; *n* = 12). Ventral bar V-shaped, with constant width: w = 2–4 (3; *n* = 13); x = 24–28 (26; *n* = 13). Haptor with 7 pairs of short hooks, each hook with erect thumb and shank comprised of 2 subunits: pair I = 11–13 (12; *n* = 13) long, pair II = 11–13 (12; *n* = 13) long, pair III = 12–14 (13; *n* = 13) long, pair IV = 13–15 (14; *n* = 13) long, pair V = 10–12 (11; *n* = 13) long, pair VI = 13–15 (14; *n* = 13) long, pair VII = 12–14 (13; *n* = 13) long. Male copulatory organ composed of irregularly shaped basal bulb and long, wavy copulatory tube with thick wall, constricted and curved approximately at proximal third, with wide terminal opening: MCO = 31–41 (37; *n* = 14); Ct = 42–47 (45; *n* = 14). Small, irregular sclerotized structure flange-like, probably part of the accessory piece, surrounds basal bulb and is considered to be the heel, He = 1–2 (1; *n* = 14). Accessory piece with proximal constrictions, distal part similar to copulatory tube in thickness, ending in a composed portion, one extremity shorter than the other, Ap = 28–35 (32; *n* = 14). Vagina non-sclerotized.

### Differential diagnosis

*Cichlidogyrus glacicremoratus* n. sp. belongs to the same group as *C. milangelnari* n. sp., *C. jeanloujustinei* n. sp., and *C. evikae* n. sp. These species share the small size of all hook pairs. *Cichlidogyrus glacicremoratus* n. sp. is similar to *C*. *vandekerkhovei* isolated from *O. boops, O. ventralis* and *O. nasuta* [23] regarding the morphology of the dorsal and ventral anchors. However, the new species is easily distinguishable from the latter by (i) the shorter dorsal bar auricles (14–18 *vs* 24–34 μm), (ii) the heel (irregular flange-like *vs* well-developed), (iii) the copulatory tube (constricted proximally, wavy with wide terminal opening *vs* narrowing distally), and (iv) the accessory piece (curved *vs* straight).

### *Cichlidogyrus rectangulus* n. sp.

- ***Type-host*** **:** *Ophthalmotilapia nasuta* (Poll & Matthes, 1962) (Fig. 1d); tribe Ectodini (Perciformes: Cichlidae).
- ***Type-locality*** **:** Magara (3°44′S, 29°19′E), Lake Tanganyika, Burundi.
- ***Type-material*** **:** Holotype: MRAC_vermes_37942. Paratypes: MRAC_vermes_37949; MRAC_vermes_37951; MNHN HEL585; SAMC-A088697.
- ***Site in host*** **:** Gills.
- ***Prevalence and intensity of infection*** **:** 75% (3/4); 1–5 monogeneans per infected host.
- ***ZooBank registration*** **:** To comply with the regulations set out in article 8.5 of the amended2012 version of the *International Code of Zoological Nomenclature* (ICZN) [36], details of the new species have been submitted to ZooBank. The Life Science Identifier (LSID) of the article is urn:lsid:zoobank.org:pub:3B9F16F6-8E3F-44F5-8D5D-B1D4A4754242. The LSID for the new name *Cichlidogyrus rectangulus* is urn:lsid:zoobank.org:act:7CFFD130-2733-46CF-984C-48482EAB2734.
- ***Etymology*** **:** The specific name “*rectangulus*” is derived from the Latin “rectangulum” which refers to the geometric shape of the heel.

### Description

**Fig. 9:**
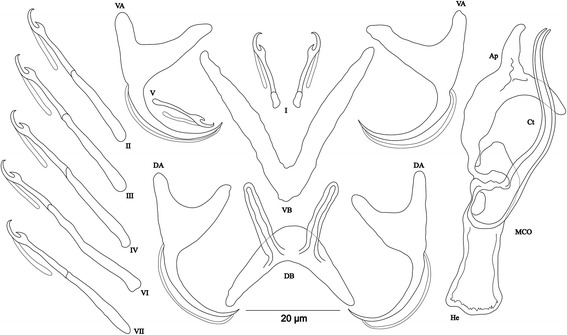
Sclerotized structures of *Cichlidogyrus rectangulus* n. sp. ex *Ophthalmotilapia nasuta*. *Abbreviations*: DA, dorsal anchor; DB, dorsal bar; VA, ventral anchor; VB, ventral bar; I-VII, hooks; MCO, male copulatory organ; He, heel; Ct, copulatory tube; Ap, accessory piece

[Based on 12 specimens fixed in GAP; Fig. 9]. Body 307–568 (513; *n* = 7) long, 109–165 (142; *n* = 8) wide at mid-body. Dorsal anchors with shaft slightly shorter than guard, blade curved in distal third, with arched point: a = 26–29 (28; *n* = 6); b = 22–26 (24; *n* = 6); c = 6–8 (7; *n* = 6), d = 8–12 (10; *n* = 6); e = 6–8 (7; *n* = 6). Dorsal bar strongly arched, thick in middle part, with straight narrow auricles: h = 12–18 (14; *n* = 6); w = 5–7 (6; *n* = 6); x = 25–29 (27; *n* = 6), y = 6–8 (7; *n* = 6). Ventral anchors with more pronounced guard than shaft (*c.*2 times length of shaft), curved blade with arched point: a = 25–27 (26; *n* = 6); b = 21–25 (22; *n* = 6); c = 6–8 (7; *n* = 6), d = 13–15 (14; *n* = 6); e = 8–12 (9; *n* = 16). Ventral bar V-shaped, with constant width: w = 4–8 (6; *n* = 7); x = 26–38 (34; *n* = 7). Haptor with 5 pairs of long hooks, hooks I and V shorter (sensu [11, 37]); each hook with erect thumb and shank comprised of 2 subunits: pair I = 17–19 (18; *n* = 6) long, pair II = 32–38 (35; *n* = 6) long, pair III = 37–41 (39; *n* = 6) long, pair IV = 38–41 (40; *n* = 6) long, pair V = 13–15 (14; *n* = 5) long, pair VI = 32–41 (36; *n* = 6) long, pair VII = 32–41 (37; *n* = 6) long. Male copulatory organ bulky, with copulatory tube beginning in ovoid bulb and relatively thick wall, S-shaped and narrower in distal portion: MCO = 57–65 (62; *n* = 9); Ct = 42–53 (47; *n* = 9). Heel long, thick, rectangular, He = 18–22 (20; *n* = 12). Accessory piece linked to basal bulb by broad connection, thick and curved in middle, with bifurcate ending: Ap = 32–36 (34; *n* = 9). Vagina non-sclerotized.

### Differential diagnosis

Based on the haptoral sclerites, *C. rectangulus* n. sp. belongs to the group of species with shorter hook pair I (pair V with larval size) and longer pairs II-IV, VI and VII (see [21]), a copulatory tube without a swollen proximal portion, and a non-sclerotized vagina (see [37]). This group includes a single species, *C. sturmbaueri* Vanhove, Volckaert & Pariselle, 2011, a parasite previously found on *O. nasuta* and *O. ventralis*. The latter was the first species of *Cichlidogyrus* hitherto described from endemic Tanganyikan cichlids displaying short hook pair I and long hook pairs II-IV, VI and VII (see [23]), and therefore *C. rectangulus* n. sp. is the second representative in the lake. *Cichlidogyrus rectangulus* n. sp. shares the host species *O. nasuta* with *C. sturmbaueri*. In addition, both species possess similarly-sized transversal bars, a similarly-shaped accessory piece, and a heel in the MCO. However, *C. rectangulus* n. sp. differs from *C. sturmbaueri* by the (i) longer dorsal anchors (26–29 *vs* 19–21 μm), (ii) longer hook pairs (almost twice as long in *C. rectangulus* n. sp. compared to *C. sturmbaueri*), (iii) size and shape of the heel (long rectangular, 18–22 μm *vs* short heel, 4–7 μm), (iv) longer copulatory tube (42–53 *vs* 34–39 μm), and (v) longer accessory piece (32–36 *vs* 24–28 μm).

### *Cichlidogyrus discophonum* n. sp.

- ***Type-host*** **:** *Aulonocranus dewindti* (Boulenger, 1899) (Fig. 1e); tribe Ectodini (Perciformes: Cichlidae).
- ***Type-locality*** **:** Nyaruhongoka (3°41′S, 29°20′E), Lake Tanganyika Burundi.
- ***Type-material*** **:** Holotype: MRAC_vermes_37945. Paratypes: MRAC_vermes_37945; MRAC_vermes_37956; SAMC-A088700.
- ***Site in host*** **:** Gills.
- ***Prevalence and intensity of infection*** **:** 33% (1/3); 1–8 monogeneans per infected host.
- ***ZooBank registration*** **:** To comply with the regulations set out in article 8.5 of the amended 2012 version of the *International Code of Zoological Nomenclature* (ICZN) [36], details of the new species have been submitted to ZooBank. The Life Science Identifier (LSID) of the article is urn:lsid:zoobank.org:pub:3B9F16F6-8E3F-44F5-8D5D-B1D4A4754242. The LSID for the new name *Cichlidogyrus discophonum* is urn:lsid:zoobank.org:act:CFC631DE-63E3-4A0C-BC20-AD2FC3AB3CDA.
- ***Etymology*** **:** The specific name “*discophonum*” is derived from the Latin “discophonum”, meaning compact disk reader, which refers to the characteristic shape of the copulatory organ.

### Description

**Fig. 10:**
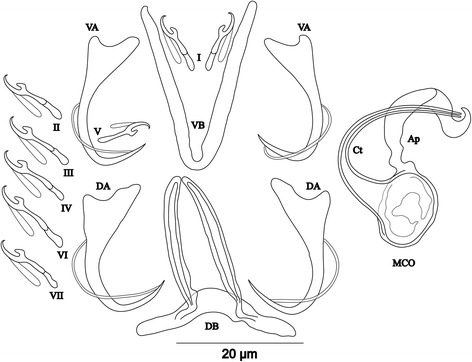
Sclerotized structures of *Cichlidogyrus discophonum* n. sp. ex *Aulonocranus dewindti*. *Abbreviations*: DA, dorsal anchor; DB, dorsal bar; VA, ventral anchor; VB, ventral bar; I-VII, hooks; MCO, male copulatory organ; He, heel; Ct, copulatory tube; Ap, accessory piece

[Based on 8 specimens fixed in GAP; Fig. 10]. Body 537–658 (612; *n* = 5) long, 88–146 (111; *n* = 6) wide at mid-body. Dorsal anchors with short shaft and more pronounced guard (*c.*2 times length of shaft) and curved blade with slightly arched point: a = 21–23 (22; *n* = 6); b = 18–21 (19; *n* = 6); c = 1–3 (2; *n* = 6); d = 4–6 (5; *n* = 6); e = 7–9 (8; *n* = 6). Dorsal bar slightly arched, thick in middle part, with blunt endings and long auricles: h = 18–20 (19; *n* = 7); w = 3–5 (4; *n* = 7); x = 22–27 (25; *n* = 7); y = 4–7 (5; *n* = 7). Ventral anchors with shorter shaft than guard and slightly arched point: a = 20–22 (21; *n* = 6); b = 18–20 (19; *n* = 6); c = 1–3 (2; *n* = 6), d = 4–6 (5; *n* = 6); e = 5–8 (7; *n* = 6). Ventral bar V-shaped: w = 2–4 (3; *n* = 7); x = 26–28 (27; *n* = 7). Haptor with 7 pairs of short hooks, hooks V with larval size (see above); each hook with erect thumb and shank comprised of 2 subunits: pair I = 10–12 (11; *n* = 12) long, pair II = 11–13 (12; *n* = 12) long, pair III = 12–14 (13; *n* = 6) long, pair IV = 13–15 (14; *n* = 6) long, pair V = 9–11 (10; *n* = 5) long, pair VI = 13–16 (15; *n* = 7) long, pair VII = 11–15 (13; *n* = 6) long. Male copulatory organ composed of long C-shaped copulatory tube, thick proximally, with large ovoid basal bulb, tapering distally: MCO = 26–34 (29; *n* = 8); Ct = 41–47 (44; *n* = 8). Heel absent. Accessory piece short, with 2 thick distinct parts, twisted distally, ending in hook, Ap = 15–22 (18; *n* = 8). Vagina non-sclerotized.

### Differential diagnosis

*Cichlidogyrus discophonum* n. sp. belongs to the same morphological group as *C. milangelnari* n. sp., *C. jeanloujustinei* n. sp., *C. evikae* n. sp. and *C. glacicremoratus* n. sp. This species is similar to *C. milangelnari* n. sp. in the morphology of the haptoral structures (ventral bar and hook pairs) and in having a MCO without a heel. However, it is easily distinguishable from *C. milangelnari* n. sp. by (i) the size of the dorsal and ventral anchors [21–22 μm (same size for both anchors) *vs* 33–36 and 32–35 μm, respectively], (ii) the copulatory tube (large ovoid basal bulb and C-shaped copulatory tube, thick proximally and tapering distally *vs* small bulb, curved at the distal third), and (iii) the accessory piece (short with two thick distinct parts, distally twisted ending in hook, 15–22 μm long *vs* thick and two superimposed parts with forked ending, 34–38 μm long). *Cichlidogyrus discophonum* n. sp. resembles *C*. *makasai*, a gill parasite of the ectodine cichlids *O. nasuta*, *O. boops* (Boulenger, 1901) and *O. ventralis* (Boulenger, 1898) [23] by the morphology of haptoral and reproductive structures: (i) small, slender dorsal and ventral anchors with shorter shaft than guard, and (ii) curved copulatory tube tapering distally. However, it can be easily distinguished from *C*. *makasai* by (i) the absence of a heel (*vs* pronounced heel in *C*. *makasai*), (ii) the length of the copulatory tube (41–47 *vs* 69–79 μm), and (iii) the shape of the accessory piece (two thick distinct parts, twisted distally with hook-like ending *vs* simple and slightly bent at distal third with spanner-like ending). *Cichlidogyrus discophonum* n. sp. is easily distinguishable from *C*. *vandekerkhovei* (also found on *O. boops, O. nasuta* and *O. ventralis* [23]) in having a shorter accessory piece with different shape (two thick distinct parts, distally twisted ending in hook, 15–22 μm *vs* straight with forked ending, one extremity shorter than the other and sometimes crossed, 24–34 μm). In addition, *C. discophonum* n. sp. lacks a heel unlike *C*. *vandekerkhovei*.

### *Cichlidogyrus pseudoaspiralis* n. sp.

- ***Type-host*** **:** *Aulonocranus dewindti* (Boulenger, 1899) (Fig. 1e); tribe Ectodini (Perciformes: Cichlidae).
- ***Type-locality*** **:** Nyaruhongoka (3°41′S, 29°20′E), Lake Tanganyika, Burundi.
- ***Type-material*** **:** Holotype: MRAC_vermes_37944. Paratypes: MRAC_vermes_37955; MNHN HEL587; SAMC-A088699.
- ***Site in host*** **:** Gills.
- ***Prevalence and intensity of infection*** **:** 33% (1/3); 1–8 monogeneans per infected host.
- ***ZooBank registration*** **:** To comply with the regulations set out in article 8.5 of the amended 2012 version of the *International Code of Zoological Nomenclature* (ICZN) [36], details of the new species have been submitted to ZooBank. The Life Science Identifier (LSID) of the article is urn:lsid:zoobank.org:pub:3B9F16F6-8E3F-44F5-8D5D-B1D4A4754242. The LSID for the new name *Cichlidogyrus pseudoaspiralis* is urn: urn:lsid:zoobank.org:act:F69F41FB-C806-40E4-99F0-7FE8BA1FBCC7.
- ***Etymology*** **:** The specific epithet is the combination of the Latin prefix “*pseudo*” and “*aspiralis*”, referring to the similarity of the new species to *C. aspiralis* n. sp. described above.

### Description

**Fig. 11:**
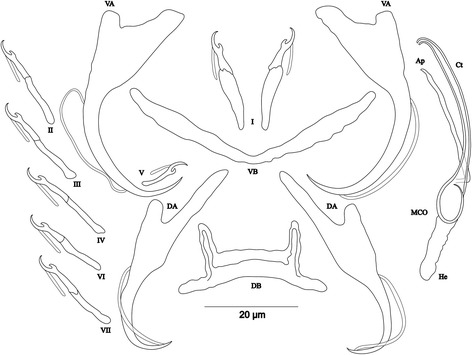
Sclerotized structures of *Cichlidogyrus pseudoaspiralis* n. sp*.* ex *Aulonocranus dewindti*. *Abbreviations*: DA, dorsal anchor; DB, dorsal bar; VA, ventral anchor; VB, ventral bar; I-VII, hooks; MCO, male copulatory organ; He, heel; Ct, copulatory tube; Ap, accessory piece

[Based on 8 specimens fixed in GAP; Fig. 11]. Body 545–714 (639; *n* = 3) long, 113–129 (121; *n* = 3) wide at mid-body. Dorsal anchors with short shaft and elongated guard (*c.*5 times length of shaft) and short, slightly bent blade and curved point: a = 37–42 (40; *n* = 3); b = 27–28 (27; *n* = 3); c = 2–4 (3; *n* = 3); d = 13–16 (15; *n* = 3); e = 6–7 (7; *n* = 3). Dorsal bar straight, thick, long with short appendages of anterior face of dorsal transverse bar: h = 6–7 (7; *n* = 3); w = 4–6 (5; *n* = 3); x = 31–32 (32; *n* = 3); y = 14–15 (14; *n* = 3). Ventral anchors with shaft shorter than guard, blade longer than in dorsal anchors, with arched point: a = 37–40 (39; *n* = 3); b = 34–37 (36; *n* = 3); c = 2–4 (3; *n* = 3); d = 9–11 (10; *n* = 3); e = 10–11 (10; *n* = 3). Ventral bar V-shaped: w = 3–4 (4; *n* = 3); x = 30–32 (31; *n* = 3). Hook pair I with well-developed shank, long in comparison with remaining pairs which are similarly short (sensu [11, 37]), pair V retains its larval size; each hook with erect thumb and shank comprised of 2 subunits: pair I = 22–24 (23; *n* = 3) long, pair II = 20–22 (21; *n* = 3) long, pair III = 21–23 (22; *n* = 3) long, pair IV = 21–24 (22; *n* = 3) long, pair V = 10–12 (11; *n* = 3) long, pair VI = 15–16 (16; *n* = 3) long, and pair VII = 17–18 (18; *n* = 3) long. Male copulatory organ beginning in ovoid bulb, with relatively long, curved and thin copulatory tube: MCO = 52–56 (54; *n* = 8); Ct = 40–43 (42; *n* = 8). Heel long, straight, He = 14–17 (16; *n* = 8). Accessory piece thin, straight, proximally with thin elbow-shaped connection to copulatory tube, Ap = 26–31 (29; *n* = 8). Vagina non-sclerotized.

### Differential diagnosis

The new species *C. pseudoaspiralis* n. sp. belongs to the group of *Cichlidogyrus* spp. characterized by a long hook pair I (pair V with larval size) and short pairs II-IV, VI and VII (see [21]), a copulatory tube without a swollen proximal portion and a non-sclerotized vagina (see [37]). This group includes *C. arfii* Pariselle & Euzet, 1995 [25]; *C. berradae* Pariselle & Euzet, 2003 [38]; *C. dionchus* Paperna, 1968 [52]; *C. halinus* Paperna, 1969 [53]; *C. muzumanii* Muterezi Bukinga, Vanhove, Van Steenberge & Pariselle, 2012 [41]; *C. nuniezi* Pariselle & Euzet, 1998 [47]; *C. papernastrema* Price, Peebles & Bamford, 1969 [54]; *C. philander* Douëllou, 1993 [55]; *C. quaestio* Douëllou, 1993 [55]; *C. reversati* Pariselle & Euzet, 2003 [38]; and *C. yanni* Pariselle & Euzet, 1996 [56]. *Cichlidogyrus pseudoaspiralis* n. sp. is similar to the new species *C. aspiralis* n. sp. described above in the morphology of the haptoral structures (hook pairs, dorsal and ventral anchors) and the relatively straight heel. However, it is easily distinguished from *C. aspiralis* n. sp. by (i) the shorter dorsal (31–32 *vs* 45–47 μm) and ventral bars (30–32 *vs* 37–40 μm), (ii) the longer copulatory tube (40–43 *vs* 20–21 μm), (iii) the longer accessory piece (26–31 *vs* 14–19 μm), and (iv) the vagina (absent in *C. pseudoaspiralis* n. sp.). Further, as in *C. aspiralis* n. sp., the dorsal bar in *C. pseudoaspiralis* n. sp. is similar to that exhibited by the monogenean species of the tylochromine cichlid hosts (see above). Thus, the new species is mainly distinguishable from *C. muzumanii* by (i) the differently sized and shaped dorsal bar (31–32 *vs* 45–62 μm), (ii) the copulatory tube (relatively long, curved and thin copulatory tube, 40–43 μm in *C. pseudoaspiralis* n. sp. *vs* penis starting in a considerable bulb, with broad and thick walled spirally coiled tube, 57–68 μm in *C. muzumanii*), (iii) the heel (14–17 *vs* 4–7 μm in *C. muzumanii*), and (iv) the accessory piece (thin elbow-shaped connection to the copulatory tube, 26–31 μm in *C. pseudoaspiralis* n. sp. *vs* not attached to the copulatory tube, 17–20 μm in *C. muzumanii*). In addition, like *C. aspiralis* n. sp., *C. pseudoaspiralis* n. sp. is easily distinguishable from *C. centesimus* by the absence of a spiral in the copulatory tube and the presence of an accessory piece (see above). Further, the new species is distinguishable from *C. casuarinus* by (i) the shorter dorsal (37–42 *vs* 52–64 μm) and ventral anchors (37–40 *vs* 47–59 μm), (ii) the shorter dorsal (31–32 *vs* 64–85 μm) and ventral bars (30–32 *vs* 54–67 μm), (iii) the hook pair I (long and well developed vs long but not thick), (iv) the shorter heel (14–17 *vs* 40–59 μm), (v) the longer and differently shaped copulatory tube (relatively long, curved and thin copulatory tube, 40–43 μm in *C. pseudoaspiralis* n. sp. *vs* straight and pointed, with distal external wall exhibiting a typical spirally coiled thickening, 34–44 μm in *C. casuarinus*), and (vi) the sclerotized vagina (present in *C. casuarinus*).

### Remarks on the diversity of the sclerotized structures in species of *Cichlidogyrus*

The new species of *Cichlidogyrus* described herein belong to three morphological groups according to the relative length of their haptoral hook pairs following Pariselle & Euzet [11], and Vignon et al. [21]. As already mentioned in the diagnoses, *C. milangelnari* n. sp. (Fig. 4), *C. jeanloujustinei* n. sp. (Fig. 5), *C. evikae* n. sp. (Fig. 6), *C. glacicremoratus* n. sp. (Fig. 8), and *C. discophonum* n. sp. (Fig. 10) belong to the group of species with short hook pairs I-IV, IV and VII. However, *C. evikae* n. sp. displays a characteristic shape of the hooks (broad thumb with a proximal protrusion). The closely related *C. aspiralis* n. sp. (Fig. 7) and *C. pseudoaspiralis* n. sp. (Fig. 11) belong to the group of species with long and well-developed hook pair I (pair V with larval size) and short pairs II-IV, VI and VII. Conversely, according to the definition of Pariselle & Euzet [11], its Tanganyikan congener *C. centesimus* possesses long and large hook pair I, and long pairs II-IV, VII and VII, a hook configuration observed in the type-species *C. arthracanthus* Paperna, 1960 described on *Coptodon zillii* (Gervais, 1848) (see [23, 24], figures and diagnosis). According to Vignon et al. [21], *C. arthracanthus* displays characteristic configuration of the hook pairs and therefore belongs to none of the major groups. Moreover, in Lake Tanganyika, *C. casuarinus* found on *B. minor* and *C. nshomboi* Muterezi Bukinga, Vanhove, Van Steenberge & Pariselle, 2012 described from *Boulengerochromis microlepis* (Boulenger, 1899) possess a characteristic hook configuration with a thin hook pair I and long pairs II-IV, VI and VII [28, 41]. *Cichlidogyrus rectangulus* n. sp. (Fig. 9) exhibits a short hook pair I and long pairs II-IV, VI and VII, with *C. sturmbaueri* hitherto as sole known representative of this morphological group in Lake Tanganyika ([23], see above). Finally, following Pariselle & Euzet [11], some other species of *Cichlidogyrus*, with their hook pairs, “escape” from the classification based on the hook configuration: we find the non-Tanganyikan species *C. nandidae* Birgi & Lambert, 1986 found on the non-cichlid host *Polycentropsis abbreviata* Boulenger, 1901 possess long hook pairs I-IV, VI and VII (pair I not large) [57], *C. kothiasi* showing well developed and long pair I with resembling pairs II-IV, VI and VII in size [51], and finally *C. chrysopiformis* Pariselle, Bitja Nyom & Bilong Bilong, 2013 from *T. bemini* Thys van der Audenaerde, 1972, with hook pair I of medium size, but not large, and short pairs II-IV, VI and VII [37].

Based on the original descriptions and the systematic review of African monogenean species published by Pariselle & Euzet [11], an overview of 111 species within *Cichlidogyrus* (including the new species described in this study) is provided (see Additional file 1: Table S1) focusing on the structural diversity of two reproductive organ features, i.e. the vagina and the heel as part of the MCO, and reviewing their presence/absence in *Cichlidogyrus* spp. First, our summary data (Additional file 1: Table S1) show that a total of 56 *Cichlidogyrus* spp. described on non-Tanganyikan cichlid hosts exhibit a sclerotized vagina. This feature has been mentioned in the original descriptions and drawings. Conversely, in 19 species of *Cichlidogyrus*, the vagina is non-sclerotized and therefore, not visible. However, a few species of *Cichlidogyrus* show a sclerotization in the vagina but only in the opening. In the last two cases, the authors did not provide any drawing or morphological characterization of the vagina. From Lake Tanganyika, we listed only five *Cichlidogyrus* spp. possessing a sclerotized vagina while the vagina of the remaining species (27 species) is non-sclerotized. Within the first haptoral group (i.e. short hook pairs I-IV, IV and VII), we listed 18 non-Tanganyikan species exhibiting a sclerotized vagina. These are *C*. *acerbus* Dossou, 1982 [49]; *C*. *amieti* Birgi & Euzet, 1983 [58]; *C*. *amphoratus* Pariselle & Euzet, 1996 [56]; *C. berrebii* Pariselle & Euzet, 1994 [51]; *C. cirratus* Paperna, 1964 [59]; *C*. *cubitus* Dossou, 1982 [49]; *C. djietoi* [46]; *C*. *giostrai* Pariselle, Bilong Bilong & Euzet, 2003 [45]; *C. karibae* Douëllou, 1993 [55]; *C*. *levequei* Pariselle & Euzet, 1996 [56]; *C*. *louipaysani* Pariselle & Euzet, 1995 [26]; *C. mvogoi* Pariselle, Bitja Nyom & Bilong Bilong, 2014 [46]; *C. njinei* Pariselle, Bilong Bilong & Euzet, 2003 [45]; *C*. *ornatus* Pariselle & Euzet, 1996 [56]; *C*. *pouyaudi* Pariselle & Euzet, 1994 [51]; *C*. *sclerosus* Paperna & Thurston, 1969 [44]; *C*. *slembroucki* Pariselle & Euzet, 1998 [47]; and *C*. *zambezensis* Douëllou, 1993 [55]. From Lake Tanganyika, a single species, *C*. *mbirizei* Muterezi Bukinga, Vanhove, Van Steenberge & Pariselle, 2012, possesses a sclerotized vagina [46]. The three Tanganyikan species, *C. casuarinus*, *C. centesimus* and *C. nshomboi*, belonging to none of the morphological groups (see above) were originally described lacking a sclerotized vagina. Later, this feature has been reported by Pariselle et al. Unlike *C. rectangulus* n. sp. and *C. sturmbaueri* showing the last hook configuration (i.e. short hook pair I and long pairs II-IV, VI and VII, see above), all *Cichlidogyrus* spp. described so far belonging to this morphological group (all non-Tanganyikan) possess a sclerotized vagina. These are *C. aegypticus* Ergens, 1981 [60]; *C. agnesi* Pariselle & Euzet, 1995 [26]; *C. anthemocolpos* Dossou, 1982 [49]; *C. bilongi* Pariselle & Euzet, 1995 [26]; *C. bonhommei* Pariselle & Euzet, 1998 [47]; *C. bouvii* Pariselle & Euzet, 1997 [40]; *C. dossoui* Douëllou, 1993 [55]; *C. douellouae* Pariselle, Bilong Bilong & Euzet, 2003 [45]; *C. ergensi* Dossou, 1982 [49]; *C. flexicolpos* Pariselle & Euzet, 1995 [26]; *C. gallus* Pariselle & Euzet, 1995 [26]; *C. gillesi* Pariselle, Bitja Nyom & Bilong Bilong, 2013 [38]; *C. guirali* Pariselle & Euzet, 1997 [40]; *C. hemi* Pariselle & Euzet, 1998 [47]; *C. kouassii* N’Douba, Thys van den Audenaerde & Pariselle, 1997 [61]; *C. legendrei* Pariselle & Euzet, 2003 [38]; *C. lemoallei* Pariselle & Euzet, 2003 [38]; *C. microscutus* Pariselle & Euzet, 1996 [56]; *C. ouedraogoi* Pariselle & Euzet, 1996 [56]; *C. paganoi* Pariselle & Euzet, 1997 [40]; *C. testificatus* Dossou, 1982 [49]; *C. thurstonae* Ergens, 1981 [60]; *C. tiberianus* Paperna, 1960 [24]; and *C. vexus* Pariselle & Euzet, 1995 [26] (see Additional file 1: Table S1).

Finally, as shown in Additional file 1: Table S1, a heel was reported, drawn and measured in most of the descriptions (70 species). Next, few papers reported the presence of the heel without measurements (19 species) or only in the drawings of the MCO (12 species). In such case, the structure of the heel was deduced and a short characterisation based on the original drawings is suggested. Moreover, seven species of non-Tanganyikan *Cichlidogyrus* are totally lacking a heel: *C. arfii*, *C. haplochromii*, *C. karibae*, *C. longicirrus*, C*. longipenis*, *C. sanseoi*, and *C. tilapiae* (see Additional file 1: Table S1); in Lake Tanganyika only *C. attenboroughi* shows this character ([16], see above and Additional file 1: Table S1). The original drawing of the non-Tanganyikan species *C. papernastrema* shows a MCO lacking a heel, while the holotype slide reveals a visible heel on the bottom of the basal bulb ([54, 39]). Furthermore, different heel sizes are sometimes found in species exhibiting a similar heel shape, i.e. *C. berradae* isolated from *Tilapia cabrae* Boulenger, 1899, *C. digitatus* and *C. yanni* from *C. zillii* which present a relatively thin and slender heel, *C. ergensi* isolated from *C. zillii* and *C. ouedraogoi* from *T. coffea* Thys van den Audenaerde, 1970 present a bean-shaped heel [37, 49, 56].

## Discussion

Monogeneans are an ideal group of organisms for studying evolutionary mechanisms because of their remarkable species richness, morphological diversity and wide distribution [62]. They developed a broad range of specialized attachment organs, probably linked with host specificity [63]. Considering the high diversity of cichlids in the Lake Tanganyika where the cichlid flocks consist of hundreds genetically and morphologically highly diverse endemic species [64], we can hypothesize a high diversity of cichlid-specific monogenean parasites, e.g. *Cichlidogyrus* spp. As already mentioned, the number of species of *Cichlidogyrus* studied and described so far remains small compared to the extraordinary diversity of their potential cichlid hosts in Lake Tanganyika.

Our investigation of five cichlid species from the Burundi coast revealed eight new *Cichlidogyrus* spp. which are described herein. Three of these cichlid species belong to tribes that were not previously investigated for the presence of monogeneans, i.e. the cyprichromine *C. microlepidotus* and the eretmodines *E. marksmithi* and *T. irsacae*; these are new host records for representatives of *Cichlidogyrus*. Three species of *Cichlidogyrus* were described from these cichlids: *C. milangelnari* n. sp. from *C. microlepidotus*, *C. jeanloujustinei* n. sp. from *E. marksmithi* and *C. evikae* n. sp. from *T. irsacae*. Several haptoral (small dorsal and ventral anchors, dorsal bar relatively short and ventral bar similar in shape and size), as well as some general copulatory organ characteristics (ovoid basal bulb prolonged into a copulatory tube with thick wall and accessory piece composed of two superimposed parts) clearly suggest an affinity between *C. jeanloujustinei* n. sp. and *C. evikae* n. sp., and therefore reflect the phylogenetic relationship between their hosts *E. marksmithi* and *T. irsacae*, both belonging to the tribe Eretmodini. Similar observations, i.e. the morphological similarity of the sclerotized parts of closely related Tanganyikan *Cichlidogyrus* spp. have been reported for tropheine hosts and host choice was clearly associated to phylogenetic relatedness of the cichlid hosts [15, 17, 42, 65].

Furthermore, the distribution of the morphologically similar new species *C. pseudoaspiralis* n. sp. and *C. aspiralis* n. sp. on *A. dewindti* and *O. nasuta*, respectively (*O. nasuta* was previously investigated for the presence of monogenean species in more southern localities in Lake Tanganyika) mirrors the relatedness between the two hosts, both belonging to the tribe Ectodini [66]. Indeed, the two new *Cichlidogyrus* spp. exhibit the same morphotype and similarities in the shape and/or size of the sclerotized structures are mainly visible in the ventral and dorsal anchors, the hook pairs and the heel in the MCO (see diagnoses and drawings). Intraspecific variability was reported in the heel length of *C. centesimus,* a species exhibiting the same morphotype as the two new species described here, i.e. *C. aspiralis* n. sp. and *C. pseudoaspiralis* n. sp. [23]. However, the shorter dorsal and ventral bars in addition to the longer MCO (longer copulatory tube and accessory piece) and non-sclerotized vagina in *C. pseudoaspiralis* n. sp., make it distinct from *C. aspiralis* n. sp.

Our study of *O. nasuta* in Burundi revealed the presence of three new monogenean species i.e. *C. aspiralis* n. sp., *C. glacicremoratus* n. sp. and *C. rectangulus* n. sp.: these were well differentiated from the four species previously described by Vanhove et al. [23] on *O. nasuta* and its congeners (see Table 1). Morphologically, *C. aspiralis* n. sp., *C. pseudoaspiralis* n. sp., *C. discophonum* n. sp. and *C. rectangulus* n. sp., share various sclerotized features with *C. centesimus*, *C*. *makasai*, *C. vandekerkhovei* and *C. sturmbaueri*. Three distinct morphotypes were distinguished. The first morphotype represented by *C. aspiralis* n. sp., *C. pseudoaspiralis* n. sp. and *C. centesimus*, is characterized mainly by a long hook pair I, a dorsal bar with short straight auricles (see diagnosis), and a MCO with a straight heel. The morphotype of *C. discophonum* n. sp., *C. glacicremoratus* n. sp., *C*. *makasai* and *C. vandekerkhovei* displays short hook pairs and a dorsal bar with long auricles. The morphotype represented by *C. rectangulus* n. sp. and *C. sturmbaueri* presents a curved dorsal bar, long hook pairs II-IV, VI and VII and a MCO with a short copulatory tube associated to an h-shaped accessory piece.

The morphological diversity within the newly described *Cichlidogyrus* spp. isolated from *Ophthalmotilapia* in Burundi and its congeners from southernmost localities (Table 1) is probably influenced by the distribution of the cichlid host in the lake and by an allopatric evolution. Indeed, it is well known now that Lake Tanganyika harbours several *Ophthalmotilapia* spp. which vary morphologically and genetically along Lake Tanganyika [67, 68]. They are well distributed along the lake [69]; Konings [70] reported five different populations of *O. nasuta* in (i) Uvira (D.R. Congo) where the holotype was caught, (ii) the Burundese shore (similar to the Uvira population), (iii) the Ubwari Peninsula (Eastern D.R. Congo) and further south to Kalemie (D.R. Congo), (iv) south of Kalemie along the western shore as far as Chimba in Zambia with a small isolated population at Cape Nangu across Cameron Bay, and (v) Zambian and Tanzanian waters which harbour the widest range of all *Ophthalmotilapia* spp. [69, 71]. Thus, it would be interesting to investigate whether the *Cichlidogyrus* fauna in Burundese *O. ventralis* follows a similar geographical variation in community composition as found for *O. nasuta*. In addition, the endemic *O. heterodonta* (Poll & Matthes, 1962) inhabiting various localities in the northern and central parts of Lake Tanganyika is the only species of this genus that has never been investigated for its parasite fauna. A study of gill ectoparasites on this cichlid host may provide additional data on the lake’s parasite species diversity. Moreover, it has been reported that *Ophthalmotilapia* spp. show low genetic diversity but present a high morphological diversity and colour plasticity, and even morphologically intermediate populations among geographically separated species were found [65]. Thus, the geographical variation of Tanganyikan *O. nasuta* probably played a role in its *Cichlidogyrus* spp. speciation and distribution. Few studies have been performed on *Cichlidogyrus* spp. infecting Tanganyikan cichlids incorporating the diversity among host populations. *Cichlidogyrus* species richness and assemblage composition in several sympatric *Simochromis diagramma* (Günther, 1894) and *Tropheus moorii* Boulenger, 1898 populations in southern Lake Tanganyika (Zambia) was studied by Grégoir et al. [72]. These authors showed seven morphologically distinct *Cichlidogyrus* spp. and significant variation of the parasite assemblages among sampling sites for *T. moorii* in contrast to *S. diagramma* which displayed a less species-rich and more homogeneous parasite fauna. Grégoir et al. [72] proposed that this difference is related to differences in dispersal capacity and hence population structure of the host species.

The present study illustrates the morphological diversity of the sclerotized parts of monogeneans in cichlids from Lake Tanganyika through the new species descriptions and the checklist of Tanganyikan and non-Tanganyikan *Cichlidogyrus* spp. Four different haptoral morphotypes of *Cichlidogyrus* species have previously been reported [11, 21, 23, 38]. Haptoral characteristics are usually used to differentiate between major lineages within *Cichlidogyrus*, whereas the morphology of the copulatory organ is more appropriate to distinguish between closely related species [17, 19, 23]. The correlation between the different hook pairs and anchors in *Cichlidogyrus* spp. was studied by Pariselle & Euzet [37] and three main groups were defined, i.e. (i) long hook pair I, short hook pair VI and long anchors, (ii) short hook pair I, short hook pair VI and medium-sized anchors, and finally, (iii) short hook pair I, long hook pair VI and small anchors. Later, Pariselle & Euzet [11] standardized the length of the hooks by dividing their total length by the total length of the hook pair V (larval size). This method was adopted to classify the length of the hook pairs i.e. “short” or “long”. Vignon et al. [21] proposed an evolutionary scenario for the configuration of the hook pairs. Morphological data in addition to phylogenetic analysis suggested that short hook pairs represent a putative primitive feature state in *Cichlidogyrus* and species later developed large hook pair I and longer pairs II-IV, VI and VII [12, 21]. Further, the study of Vignon et al. [21] allowed to classify for instance *C. kothiasi* within the group of species exhibiting short hook pairs and *C. nandidae* within the group possessing large pair I and short pairs II-IV, VI and VII. On the other hand, some Tanganyikan and non-Tanganyikan species (see above), with their “new” hook configurations, were not yet described, and therefore, not included. Therefore, we suggest that there are more than four previously reported haptoral groups. Moreover, due to the incomplete taxonomic coverage, it is still not possible to fully elucidate the evolution of the different haptoral configurations in *Cichlidogyrus* spp. It would be interesting to re-investigate the structural diversity of the hook pairs in *Cichlidogyrus* spp. and identify the exact “borders” between the haptoral groups.

In addition to the haptoral sclerites, species of *Cichlidogyrus* described so far can be clustered based on the vagina being sclerotized or non-sclerotized. In *Cichlidogyrus* spp. from Lake Tanganyika the vagina is sclerotized or not (see above). *Cichlidogyrus pseudoaspiralis* n. sp. isolated from Burundese *O. nasuta* exhibits a sclerotized vagina unlike the remaining new *Cichlidogyrus* species described herein. In fact, most Tanganyikan *Cichlidogyrus* spp. exhibit a non-sclerotized vagina (see above and Additional file 1: Table S1). On the other hand, a comparison of *Cichlidogyrus* spp. based on the vagina and the length of hook pairs revealed that only two Tanganyikan representatives of the group of species with short hooks I and long pairs II-IV, VI and VII, lack a sclerotized vagina. Our results shed light on the necessity to elucidate the evolutionary scenarios and the significance of the sclerotization in the vagina in *Cichlidogyrus* spp. It would be interesting to analyse whether there is a correlation between the reproductive organs (presence/absence of the sclerotized vagina) and the haptoral sclerites (morphology of the hook pairs).

Examination of all original drawings and descriptions of *Cichlidogyrus* spp. allowed us to highlight the high diversity in the heel structure (Additional file 1: Table S1). The descriptions of the type-species, i.e. *C. arthracanthus* on *C. zillii* reported a sclerotized structure associated with the copulatory tube, different from the accessory piece and the auxiliary plate [24, 47]. In fact, the sclerotized portion considered as a heel is part of the accessory piece [18]. This feature is absent in two of the newly described species, *C. milangelnari* n. sp. and *C. discophonum* n. sp., representing the second record of *Cichlidogyrus* spp. parasitizing Tanganyikan cichlids that lack heel, the first being *C. attenboroughi* from the benthochromine *B. horii* [16]. A clear example of morphologically related *Cichlidogyrus* spp. in endemic Tanganyikan cichlids is the long straight heel present in *C. casuarinus, C. centesimus*, *C. aspiralis* n. sp., *C. pseudoaspiralis* n. sp. and *C. nshomboi*. The characteristic shape of the heel in addition to the spirally-coiled wall of the MCO in *Cichlidogyrus* spp. infecting Bathybatini (*C. casuarinus*), Ectodini (*C. centesimus*, absent in *C. aspiralis* n. sp. and *C. pseudoaspiralis* n. sp., see above) and Boulengerochromini (*C. nshomboi*) are found exclusively in these species [16, 23, 28, 41].

## Conclusions

It is too early for conclusions about the role of host-specificity in Lake Tanganyika due to limited data on ectoparasite monogeneans in this system. Further studies to investigate cichlid fishes in the lake for parasites belonging to *Cichlidogyrus* spp. are necessary. The high morphological diversity of haptoral structures and reproductive organs of the new species described herein and other species identified so far confirms the existence of various lineages of *Cichlidogyrus* in Lake Tanganyika. However, further morphological studies and molecular data are needed to elucidate their origin and evolutionary history.

## Additional file

Additional file 1: Table S1. Overview of the vagina and the heel structures of African *Cichlidogyrus* species. (DOCX 100 kb)

## Abbreviations

MCO: Male copulatory organ
