# Supplementary material for: Underexplored diversity of gill monogeneans in cichlids from Lake Tanganyika: eight new species of Cichlidogyrus Paperna, 1960 (Monogenea: Dactylogyridae) from the northern basin of the lake, with remarks on the vagina and the heel of the male copulatory organ
Source: Parasit Vectors. 2017 Dec 2;10:591. doi: 10.1186/s13071-017-2460-6 (PMC5712084; doi:10.1186/s13071-017-2460-6)
Supplement: Additional file 1: Table S1. — Overview of the vagina and the heel structures of African Cichlidogyrus species. (DOCX 100 kb) [file 13071_2017_2460_MOESM1_ESM.docx]

**Additional file 1: Table S1.** Overview of the vagina and the heel structures of African Cichlidogyrus spp.

♠ vagina/heel mentioned in the original description and/or drawings, measurements are given. ♥ vagina/heel mentioned in the original description and/or drawings, no measurements are given. ♣ heel mentioned only on drawings, a description is suggested. ♦ original description in foreign language (French), a translation is included.

| **Parasite species** | | **Type-host** | **Type-locality** | **Remark on the vagina structure** | **Remark on the heel structure** | | **Reference** |
| --- | --- | --- | --- | --- | --- | --- | --- |
|  | **Non-Tanganyikan *Cichlidogyrus* species** | | | | |  |  |
| 1. *Cichlidogyrus acerbus*   Dossou, 1982 | | *Sarotherodon melanotheron*  Rüppell, 1852 | Ouémé and Couffo  (Benin) | “Le vagin faiblement sclérifié est un tube simple de 20-22 µm de longueur sur 2-3 d’épaisseur”; “Vagina weakly sclerified is a simple tube of 20-22 µm long and 2-3 wide” ♦♠ | “La pièce accessoire présente deux parties. La première proximale est un ruban arqué de 28-30 µm de longueur sur 2-3 µm d’épaisseur attaché à l’ampoule basale du pénis”; “Arched ribbon proximally, He= 28-30 long and 2-3 µm wide linked to the basal bulb of the penis” ♦♠ | | [1]; p. 315, 316 |
| 1. *Cichlidogyrus aegypticus*   Ergens, 1981 | | *Coptodon zillii*  (Gervais, 1848) | Cairo, Nile River (Egypt) | “Vagina armor tubular, almost in the middle regularly widened, measuring 24-26 in length” ♠ | “Copulatory complex consisting of basic portion, branched supporting portion” ♥ | | [2]; p. 211 |
| 1. *Cichlidogyrus agnesi*   Pariselle & Euzet, 1995 | | *Coptodon guineensis*  (Günther, 1862) | Tiassalé, Bandama River (Ivory Coast) | “Vagina with thickened sclerotized lining, characteristic sickle shape, V = 67.8 ± 3.2 (62-72), v = 4.3 ± 0.4 (3-4)” ♠ | “Ovoid thickened auxiliary plate with numerous roughly aligned tubercles, always present near distal region of copulatory complex, L = 19.9 ± 1.5 (17-23), l = 14.6 ± 1.1 (12-16)” ♠ | | [3]; p. 190 |
| 1. *Cichlidogyrus albareti*   Pariselle & Euzet, 1998 | | *Tilapia brevimanus*  Boulenger, 1911 | La Ramié, Bourouma River (Guinea) | “Thin vagina with funnel shaped beginning: Vg = 56 ± 7.7 (43-69) long, 1 ± 0.2 (1-2) in diameter at the middle” ♠ | “Heel variable in length, He = 8 ± 3.6 (2-16)” ♠ | | [4]; p. 276, 277 |
| 1. *Cichlidogyrus amieti*   Birgi & Euzet, 1983 | | *Aphyosemion cameronense*  (Boulenger, 1903) | Kala, Zamacoe (Cameroon) | “Le vagin ventral s'ouvre sur légèrement à droite du plan médian, légèrement sclérifié, 65-70 long aboutit à un petit réceptacle séminal situé en avant de l'ovaire”; “ventral vagina opens slightly to the right of the median, slightly sclerified, 65-70 long leads to a small seminal receptacle located anteriorly” ♦♠ | “Large heel on the bulb and associated to the accessory piece” ♣ | | [5]; p. 102, 103 |
| 1. *Cichlidogyrus amphoratus*   Pariselle & Euzet, 1996 | | *Tilapia louka*  Thys van den Audenaerde, 1969 | La Ramié, Bourouma River (Guinea) | “Thin vagina, S-shaped: L = 23 ± 1.2 (20-25), l = 2 ± 0.2 (1-2)” ♠ | “Large sub-circular heel, He = 7 (5-8)” ♠ | | [6]; p. 115 |
| 1. *Cichlidogyrus anthemocolpos*   Dossou, 1982 | | *Coptodon zillii*  (Gervais, 1848) | Ouémé (Benin) | “Le vagin est un tube de 13 µm de longueur, sinieux débutant par un élargissement à rayons mesuant 10 sur 20 µm. Une telle plaque ne se trouve chez aucun *Cichlidogyrus*”; “Tubular and sinuous vagina of 13 µm long beginning with a beam widening of 10 by 20 µm. This feature has never been found on any Cichlidogyrus” ♦♠ | “Le coude est recouvert d’un épaississement sclérifié formant un talon”; “The elbow is covered with a sclerotized thickening forming a heel” ♦♥ | | [1]; p. 308, 309 |
| 1. *Cichlidogyrus arfii*   Pariselle & Euzet, 1995 | | *Pelmatochromis buettikoferi*  (Steindachner, 1894) | Konkouré à Koleya (Guinea) | “L’ouverture vaginale est marquee par une épaisse sclérification irrégulière”; “vagina opening is marked by a thick irregular sclerotization” ♦♥ | **No heel** | | [7]; p. 205 |
| 1. *Cichlidogyrus arthracanthus*   Paperna, 1960 | | *Coptodon zillii*  (Gervais, 1848) | Fish Culture Research Station at Dor (Israel) | “The vagina opens to the left of the middle of the body, its wall being prominently thickened at the extremity” ♥ | “An ovoid, flattened plate is attached to one side of the funnel, while the accessory piece is attached to the opposite side” ♥ | | [8]; p. 4, 5 |
| 1. *Cichlidogyrus berminensis*   Pariselle, Bitja Nyom & Bilong Bilong, 2013 | | *Tilapia bemini*  Thys van den Audenaerde, 1972 | Lake Bermin (Cameroon) | **Vagina non-sclerotized** | “Penis starting as a bulb, a marked narrow heel with rounded ending, He = 5 (4-6)” ♠ | | [9]; p. 81 |
| 1. *Cichlidogyrus berradae*   Pariselle & Euzet, 2003 | | *Tilapia cabrae*  Boulenger, 1899 | Lake Cayo, Kabinda  (R. Congo) | **Vagina non-sclerotized** | “Long thin heel, He = 36 ± 1.3 (33-37)” ♠ | | [10]; p. 196, 198 |
| 1. *Cichlidogyrus berrebii*   Pariselle & Euzet, 1994 | | *Tylochromis jentinki*  (Steindachner, 1894) | Ebrié lagoon  (Ivory Coast) | “vagina opening is marked by a thick irregular sclerotization” ♥ | “Massive proximal heel” ♥ | | [11] |
| 1. *Cichlidogyrus bifurcatus*   Paperna, 1960 | | *Astatotilapia flaviijosephi*  (Lortet, 1883) | Sea of Galilee (Israel) | **Vagina non-sclerotized** | “An ovoid flattened plate is attached to one side of the funnel” ♥ | | [8] ; p. 13 |
| 1. *Cichlidogyrus bilongi*   Pariselle & Euzet, 1995 | | *Coptodon guineensis*  (Günther, 1862) | Tiassalé, Bandama River (Guinea) | “Vagina with sclerotized lining, forms sickle with long shaft, V = 43.7 ± 1.9 (39-47), v = 5.6 ± 0.6 (4-6)” ♠ | “Large semi-circular expansion at bend of accessory piece, He = 7.6 ± 0.8 (6-10)” ♠ | | [3]; p. 191, 192 |
| 1. *Cichlidogyrus bonhommei*   Pariselle & Euzet, 1998 | | *Tilapia buttikoferi*  (Hubrecht, 1881) | Katonga, Little Scarcies River (Sierra Leone) | “Looped vagina: Vg = 19 ± 2.8 (15-23) long and 3 ± 0.5 (2-4) in diameter” ♠ | “Heel poorly developed, He = 1 ± 0.4 long” ♠ | | [4]s; p. 279, 280 |
| 1. *Cichlidogyrus bouvii*   Pariselle & Euzet, 1997 | | *Sarotherodon occidentalis*  (Daget, 1962) | La Ramié, Bourouma River (Guinea) | “Short conical vagina, curved in middle: V = 9 ± 1.4 (7-12), v = 3 ± 0.4 (2-4)” ♠ | “Ovoid heel, He = 6 ± 0.5 (5-8)” ♠ | | [12]; p. 223, 224 |
| 1. *Cichlidogyrus chrysopiformis*   Pariselle, Bitja Nyom & Bilong Bilong, 2014 | | *Tylochromis sudanensis*  Daget, 1954 | Cross River at Mamfé (Cameroon) | “Short, straight and slightly ringed vagina: x = 5 (4-6, 19), w = 2 (2-3, 19)” ♠ | “Penis starting in a bulb with developed flared heel, He = 9 (7-11, 20)” ♠ | | [13]; p. 262, 263 |
| 1. *Cichlidogyrus cirratus*   Paperna, 1964 | | *Sarotherodon galilaeus*  (Linnaeus, 1758) | Off-shore of Lake Tiberias (Israel) | “Vagina armor in form of fine, long tube” ♥ | “Rounded funnel to which two 16 µ and 56 µ long accessory pieces are attached” ♠ | | [14]; p. 5, 17; [2]; p. 207 |
| 1. *Cichlidogyrus consobrini*   Jorissen, Pariselle & Vanhove, 2017 | | *Sargochromis mellandi* (Boulenger, 1905) | Kipopo INERA aquaculture station  (D.R. Congo) | **Vagina non-sclerotized** | “A rectangular heel is attached to the side of the basal bulb” ♠ | | [15]; p. 5, 7 |
| 1. *Cichlidogyrus cubitus*   Dossou, 1982 | | *Coptodon zillii*  (Gervais, 1848) | Ouémé (Benin) | “Le vagin est un tube très étroit, filiforme, légèrement arqué, de 40 µm de longeur”; “Vagina is a narrow tube, filiform, slightly arched, of 40 µm long” ♦♠ | “Long pénis débute par une base renflée piriforme, coiffée d’une sclérification épaisse”; “Thick sclerotization covers the basal bulb” ♦♥ | | [1]; p. 307 |
| 1. *Cichlidogyrus dageti*   Dossou & Birgi, 1984 | | *Hemichromis fasciatus*  Peters, 1857 | No type locality mentioned | “L’ouverture vaginale sub-médiane ventrale donne accès à un large canal sclérifié de 20 à 25 µm de longueur, relié par un étroit canalcule au réceptacle séminal antéro-ovarien”; “The ventral and subtermianl vagina opening in prolonged into a sclerified duct of 20-25 µm long, linked to a narrow duct to the antero-ovarian seminal receptacle” ♦♠ | “L’ampoule basale porte un fort talon sclérifié de 20 µm de longueur”; “The basal bulb has a strong sclerotized heel, He = 20 µm” ♦♠ | | [16]; p. 107, 108 |
| 1. *Cichlidogyrus digitatus*   Dossou, 1982 | | *Coptodon zillii*  (Gervais, 1848) | Ouémé (Benin) | “Le vagin débute par une ouverture submédio-ventrale et se poursuit par un étroit canal de 2 µm qui s’élargit brusquement en se couchant en forme de U. il mesure environ 22 µm de longueur”; “Vagina begininig with a submedio-ventral opening which continues through a narrow duct of 2-μm, that expands abruptly into a U-shape. It measures about 22 μm long” ♦♠ | “Le pénis débute par un petit renflement en ampoule portant à son extrémité une masse sclérifiée en forme de talon caractéristique”; “Sclerotized mass bean-shaped at the end of the basal bulb” ♦♥ | | [1]; p. 304, 305 |
| 1. *Cichlidogyrus dionchus*   Paperna, 1968 | | *Sarotherodon galilaeus*  (Linnaeus, 1758) | Mamahuma stream, Acca (Ghana) | **Vagina non-sclerotized** | “Thin walled funnel with a small lateral projection” ♥ | | [17]; p. 89, 91 |
| 1. *Cichlidogyrus djietoi*   Pariselle, Bitja Nyom & Bilong Bilong, 2014 | | *Tylochromis sudanensis*  Daget, 1954 | Cross River at Mamfé (Cameroon) | “Short, arched and funnel shaped vagina, x = 10 (7-13, 10), w = 2 (2-3, 10)” ♠ | “Penis starting in a bulb with developed heel, He = 7 (6-9, 10)” ♠ | | [13]; p. 263, 264 |
| 1. *Cichlidogyrus dossoui*   Douëllou, 1993 | | *Coptodon rendalli*  (Boulenger, 1897) | Kariba Lake (Zimbabwe) | “Vagina well sclerotized” ♥ | “Oval sclerotization on a side of the elongated basal bulb” ♣ | | [18]; p. 174, 176 |
| 1. *Cichlidogyrus douellouae*   Pariselle, Bilong Bilong & Euzet, 2003 | | *Sarotherodon galilaeus*  (Linnaeus, 1758) | Mékrou River, “W” National Park (Niger) | “Short and well-sclerotized vagina, bent at middle: V = 14 (12-17), v = 5 (3-7)” ♠ | “Very large heel, He = 8 (7-10)” ♠ | | [19]; p. 205 |
| 1. *Cichlidogyrus dracolemma*   Řehulková, Mendlová & Šimková, 2013 | | *Hemichromis letourneuxi*  Sauvage, 1880 | Niokolo Koba River (Senegal) | “Vagina dextroventral, with circular superficial sclerotization around distal opening of vagina duct, 17 (15-18) × 16 (15-17); vagina duct is S-shaped before it joins the seminal receptacle; total curved length 54 (46-62)” ♠ | “Copulatory tube surrounded by laterally enlarged auricle-like flange (probably basal part of the accessory piece)” ♥ | | [20]; p. 1402 |
| 1. *Cichlidogyrus ergensi*   Dossou, 1982 | | *Coptodon zillii*  (Gervais, 1848) | Ouémé (Benin) | “Le vagin est un tube simple, sinueux, à paroi légèrement sclérifiée, de 20 µm de longueur”; “Vagina is simply tubular, sinuous with slightly sclerified wall, 20 µm long” ♦♠ | “La pièce accessoire débute par une bosse sclérifiée, en forme de haricot, accolée à l’ampoule basale fusiforme”; “Sclerotized bump bean-shaped linked to a fusiform bulb” ♦♥ | | [1]; p. 302, 303 |
| 1. *Cichlidogyrus euzeti*   Dossou & Birgi, 1984 | | *Hemichromis fasciatus*  Peters, 1857 | Mouanko (Cameroon) | “Le vagin est un tube étroit où nous distinguons deux parties: celle qui suit l’ouverture est enroulée en spirale étroite, la seconde siniueuse, aboutit à un réceptacle seminal globuleux antéro-ovarien”; “Vagina is narrow tube with two parts: spirally coiled proximally and sinuous prolongated into a globular and antero-ovarian seminal receptacle” ♦♥ | “Le cirre tubulaire débute par une ampoule basale bordée par une sclérification en croissant”; “Basal bulb surrounded by a croissant-like sclerotization” ♦♥ | | [16]; p. 107, 108 |
| 1. *Cichlidogyrus falcifer*   Dossou & Birgi, 1984 | | *Hemichromis fasciatus*  Peters, 1857 | No type locality mentioned | “Le vagin faiblement sclérifié, à ouverture submédiane, est un tube étroit à son début mais qui s’élargit en formant un démi cercle”; “Vagina weakly sclerified with subtermial opening, beginning by a narrow tube which expands forming a semicircle” ♦♥ | “Une large expansion de 5 µm de long forme un talon sur l’ampoule basale”; “Wide expansion of 5 µm long forms a heel on the basal bulb” ♦♠ | | [16]; p. 105, 106 |
| 1. *Cichlidogyrus flexicolpos*   Pariselle & Euzet, 1995 | | *Coptodon guineensis*  (Günther, 1862) | Tiassalé, Bandama River (Ivory Coast) | “Long tubular vagina, V = 66.2 ± 3.2 (31-42), lightly sclerotized sinuous and folded in the middle” ♠ | “Sclerotized part linked to the accessory piece on a side of the basal bulb” ♣ | | [3]; p. 194 |
| 1. *Cichlidogyrus fontanai*   Pariselle & Euzet, 1997 | | *Sarotherodon occidentalis*  (Daget, 1962) | La Ramié, Bourouma River (Guinea) | **Vagina non-sclerotized** | “Thin narrow heel” ♥ | | [12]; p. 224, 225 |
| 1. *Cichlidogyrus gallus*   Pariselle & Euzet, 1995 | | *Coptodon guineensis*  (Günther, 1862) | Tiassalé, Bandama River (Ivory Coast) | “Vagina slightly sinuous, with thick lining, V = 32.7 ± 2.2 (25-36), v = 5 ± 0.6 (3-6)” ♠ | “Large semi-circular expansion on a side of the basal bulb” ♣ | | [3]; p. 192, 193 |
| 1. *Cichlidogyrus gillesi*   Pariselle, Bitja Nyom & Bilong Bilong, 2013 | | *Coptodon guineensis*  (Günther, 1862) | Lake Ossa (Cameroon) | “Short, S-shaped vagina with thick and wrinkled wall: x = 17 (12-29), w = 3 (2-3)” ♠ | “Penis starting perpendicularly as a large ovoid bulb with a very large trapezoid heel, He = 13 (10-16, 8)” ♠ | | [9]; p. 82, 83 |
| 1. *Cichlidogyrus giostrai*   Pariselle, Bilong Bilong & Euzet, 2003 | | *Sarotherodon caudomarginatus*  (Boulenger, 1916) | Tondon Road, Badi River (Guinea) | “Long, thin vagina with large aperture, V = 70 (62-78), v = 2 (1-2)” ♠ | “Developed heel, He = 6 (3-7)” ♠ | | [19]; p. 206 |
| 1. *Cichlidogyrus guirali*   Pariselle & Euzet, 1997 | | *Sarotherodon occidentalis*  (Daget, 1962) | La Ramié, Bourouma River (Guinea) | “Short, C-shaped vagina: V = 17 ± 2.1 (14-21), v = 4 ± 0.4 (4-5)” ♠ | “Poorly developed heel, He = 3 ± 0.4 (3-4)” ♠ | | [12]; p. 226 |
| 1. *Cichlidogyrus halinus*   Paperna, 1969 | | *Sarotherodon melanotheron*  (Rüppell, 1852) | Peshs lagoon, East of Accra (South Ghana) | **Vagina non-sclerotized** | “Well developed heel with rounded ending” ♣ | | [21], p. 860 |
| 1. *Cichlidogyrus halli*   (Price & Kirk, 1967) | | *Oreochromis shiranus*  Boulenger, 1897 | Fort Johnston, Upper Shire River (Malawi) | “**Vagina not observed** with absolute certainty, but a **non-sclerotized vagina atrium** appears top open ventrally in left body half” ♥ | “Cirrus arises from a moderately expanded base which is attached a striated plate” ♥ | | [22]; p. 139, 140;  [15], p. 9 |
| 1. *Cichlidogyrus haplochromii*   Paperna & Thurston, 1969 | | *Haplochromis guiarti*  (Pellegrin, 1904) | Jinja (Uganda) | **Vagina non-sclerotized** | **No heel** | | [23]; p. 19, 20 |
| 1. *Cichlidogyrus hemi*   Pariselle & Euzet, 1998 | | *Tilapia brevimanus*  Boulenger, 1911 | La Ramié, Bourouma River (Guinea) | “Vagina short, straight and ringed: Vg = 10 ± 1.5 (6-12) long and 2 ± 0.4 (1-3) in diameter” ♠ | “Heel, He = 7 (5-9)” ♠ | | [4]; p. 276, 278 |
| 1. *Cichlidogyrus inconsultans*   Birgi & Lambert, 1987 | | *Polycentropsis abbreviata*  Boulenger, 1901 | Mouanko (Cameroon) | “Le vagin débute par une ouverture ovalaire sclérifié de 10 µm de diamètre, lui fait suite un tube de 100-120 µm de longueur qui aboutit à un vaste réservoir seminal”; “Vagina beginning by sclerified oval opening of 10 µm of diameter, prolonged into a tube of 100-120 µm long leading to a wide seminal reservoir” ♦♠ | “Le cirre débute par l’ampoule basale soudée à l’extrémité élargie de la pièce accessoire”; “Enlarged part of the accessory piece linked to the basal bulb” ♦♠ | | [24]; p. 526, 527 |
| 1. *Cichlidogyrus karibae*   Douëllou, 1993 | | *Sargochromis codringtonii*  (Boulenger, 1908) | Kariba Lake (Zimbabwe) | “Vagina distinguishable, assuming shape of short, narrow tube” ♥ | **No heel** | | [18]; p. 177, 178 |
| 1. *Cichlidogyrus kothiasi*   Pariselle & Euzet, 1994 | | *Tylochromis jentinki*  (Steindachner, 1894) | Ebrié lagoon (Ivory Coast) | “vaginal pore funnel shaped, weakly sclerified” ♥ | “Long thin heel, He = 7.5 (7-9)” ♠ | | [11]; p. 233, 234 |
| 1. *Cichlidogyrus kouassii*   N’Douba, Thys van den Audenaerde & Pariselle, 1997 | | *Coptodon guineensis*  (Günther, 1862) | Bakro, Ayamé Lake  (Ivory Coast) | “Le vagin cylindrique à paroi mince, et le plus souvent replié en S. Il mesure 31 ± 3.7 (22-43) de longueur et 1 ± 0.3 (1-2) de largeur”; “Cylindrical vagina with thin walls, often folded in S, 31 ± 3.7 (22-43) long and 1 ± 0.3 (1-2) wide” ♦♠ | “Large ampoule basale ovoïde présentant un talon bien développé mesurant 6 ± 1.1 (5-10)”; “Well-developed heel, He = 6 ± 1.1 (5-10)” ♦♠ | | [25]; p. 431, 432 |
| 1. *Cichlidogyrus lagoonaris*   Paperna, 1969 | | *Sarotherodon melanotheron*  Rüppell, 1852 | Peshi lagoon  (South Ghana) | “Le vagin, très faiblement sclérifié, est un tube étroit de 12-13 µm de longueur“; “Vagina weakly sclerified, is a narrow tube of 12-13 µm long“ ♦♠ | “Reduced heel” ♣ | | [21]; p. 860, 862; [1]; p. 319 |
| 1. *Cichlidogyrus legendrei*   Pariselle & Euzet, 2003 | | *Tilapia cabrae*  Boulenger, 1899 | Cayo Lake, Kabinda  (R. Congo) | “Slightly arched, thick-walled vagina: V = 26 ± 2.7 (21-31), v = 5 ± 0.6 (4-6)” ♠ | “Ovoid basal bulb and heel, He = 5 ± 0.3 (4-6)” ♠ | | [10]; p. 198, 199 |
| 1. *Cichlidogyrus lemoallei*   Pariselle & Euzet, 2003 | | *Tilapia cabrae*  Boulenger, 1899 | Mouth of the Bas Kouilou River (R. Congo) | “Thin, long, slightly sinuous vagina: V = 50 ± 4 (41-58), v = 2 ± 0.4 (2-3)” ♠ | “A thin heel, 3 ± 0.8 (2-5)” ♠ | | [10]; p. 198, 199 |
| 1. *Cichlidogyrus levequei*   Pariselle & Euzet, 1996 | | *Tilapia coffea*  Thys van den Audenaerde, 1970 | Niambala, Oulé River  (Guinea) | “Short, thin vagina: V = 15 ± 1.4 (12-18), v = 2 ± 0.3 (1-2)” ♠ | ”Short heel, 1 ± 0.3 (1-2)” ♠ | | [6]; p. 121 |
| 1. *Cichlidogyrus longicirrus*   Paperna, 1965 | | *Hemichromis fasciatus*  Peters, 1857 | New Tafo (Ghana) | “The vagina is located on the left side of the body” ♥ | **No heel** | | [26]; p. 108, 111 |
| 1. *Cichlidogyrus longipenis*   Paperna & Thurston, 1969 | | *Astatoreochromis alluaudi*  Pellegrin, 1904 | Jinja (Uganda) | **Vagina non-sclerotized** | **No heel** | | [23]; p. 28, 29 |
| 1. *Cichlidogyrus louipaysani*   Pariselle & Euzet, 1995 | | *Coptodon guineensis*  (Günther, 1862) | Rice fields, Koba (Guinea) | “Vagina sinuous, V = 50.2 ± 3.8 (44-58)” ♠ | “Straight heel on basal bulb” ♥ | | [3]; p. 195, 196 |
| 1. *Cichlidogyrus microscutus*   Pariselle & Euzet, 1996 | | *Coptodon guineensis*  (Günther, 1862) | Loufoualéba Lake  (R. Congo) | “Short vagina, slightly S-shaped, with sclerotized plate at aperture: V = 19 ± 1.9 (13-22), v = 4 ± 0.4 (3-5)” ♠ | “Heel, He = 5 ± 0.6 (4-6)” ♠ | | [6]; p. 113, 114 |
| 1. *Cichlidogyrus mvogoi*   Pariselle, Bitja Nyom & Bilong Bilong, 2014 | | *Sarotherodon mvogoi*  (Thys van den Audenaerde, 1965) | Ayos, Nyong River  (Cameroon) | “Very long and thin spirally coiled v (double pitch), no valuable length could be taken” ♥ | “Penis starting in a bulb with marked heel, He = 8 (6-10, 24)” ♠ | | [13]; p. 260 |
| 1. *Cichlidogyrus nageus*   Řehulková, Mendlová & Šimková, 2013 | | *Sarotherodon galilaeus*  (Linnaeus, 1758) | Niokolo Koba River  (Senegal) | **Vagina non-sclerotized** | “Base with marginal flap bilaterally expending into two auricle-like lobes” ♥ | | [20]; p. 1405, 1406 |
| 1. *Cichlidogyrus nandidae*   Birgi & Lambert, 1986 | | *Polycentropsis abbreviata*  Boulenger, 1901 | Mouankou (Cameroon) | “L’ouverture vaginale est médio-ventrale. Le vagin débute par une ouverture circulaire de 10 × 12 µm, lui fait un tube faiblement sclérifié de 105 µm qui aboutit à un petit réceptacle séminale situant en avant de l’ovaire”; “Medio-ventral vagina opening beginning by a circular opening of 10 × 12 µm prolonged into a tube weakly sclerified of 105 µm, which leads to a small seminal receptacle anterior to the ovary” ♦♠ | “Heel on the top of the basal bulb associated to the accessory piece” ♦♣ | | [27], p. 523, 524 |
| 1. *Cichlidogyrus njinei*   Pariselle, Bilong Bilong & Euzet, 2003 | | *Sarotherodon galilaeus*  (Linnaeus, 1758) | Edéa, Sanaga River (Cameroon) | “Vagina long and sinuous: V = 64 (62-68), v = 2 (2-3)” ♠ | “Large heel, He = 8 (7-10)” ♠ | | [19]; p. 207, 208 |
| 1. *Cichlidogyrus nuniezi*   Pariselle & Euzet, 1998 | | *Tilapia cessiana*  Thys van den Audenaerde, 1968 | Toyebli, Nipoué River (Ivory Coast) | **Vagina non-sclerotized** | “Heel, He = 4 (4-5)” ♠ | | [4]; p. 278, 279 |
| 1. *Cichlidogyrus ornatus*   Pariselle & Euzet, 1996 | | *Coptodon zillii*  (Gervais, 1848) | Baoulé River (Ivory Coast) | “Thin, sinuous vagina, notable for its large, hemispherical auricle: V = 33 ± 2.9 (29-37), v = 2 ± 0.3 (2-3)” ♠ | “Heel, He = 5 (4-6)” ♠ | | [6]; p. 116 |
| 1. *Cichlidogyrus ouedraogoi*   Pariselle & Euzet, 1996 | | *Tilapia coffea*  Thys van den Audenaerde, 1970 | Niambala, Oulé River  (Guinea) | “Vagina bent at right-angle (L-shaped): V = 21 ± 2.5 (17-25), v = 4 ± 0.3 (3-4)” ♠ | “Heel, He = 5 (4-5)” ♠ | | [6]; p. 121, 121 |
| 1. *Cichlidogyrus paganoi*   Pariselle & Euzet, 1997 | | *Sarotherodon occidentalis*  (Daget, 1962) | La Ramié, Bourouma River (Guinea) | “Vagina large, straight: V = 21 ± 2.7 (17-28), v = 5 ± 0.9 (4-8) in diameter” ♠ | “Heel, He = 6 (5-8)” ♠ | | [12]; p. 227, 228 |
| 1. *Cichlidogyrus papernastrema*   Price, Peebles & Bamford, 1969 | | *Tilapia sparrmanii*  Smith, 1840 | Natal (R. South Africa) | **Vagina non-sclerotized** | No heel in the original drawing but well pronounced one is present on the MCO of the type material | | [28]; p. 119, 121;  [15], p. 10 |
| 1. *Cichlidogyrus philander*   Douëllou, 1993 | | *Pseudocrenilabrus philander*  (Weber, 1897) | Kariba Lake (Zimbabwe) | **Vagina non-sclerotized** | “Short straight heel” ♣ | | [18]; p. 183, 184 |
| 1. *Cichlidogyrus pouyaudi*   Pariselle & Euzet, 1994 | | *Tylochromis jentinki*  (Steindachner, 1895) | Ebrié lagoon (Ivory Coast) | “vagina opening lightly sclerotized” ♥ | “Long proximal heel, He = 4.9 (4-6)” ♠ | | [11]; p. 232 |
| 1. *Cichlidogyrus quaestio*   Douëllou, 1993 | | *Tilapia rendalli*  (Boulenger, 1897) | Kariba Lake (Zimbabwe) | **Vagina non-sclerotized** | “Copulatory tube slightly curved with wide base capped with regular, almost square portion” ♥ | | [18], p. 181, 182 |
| 1. *Cichlidogyrus reversati*   Pariselle & Euzet, 2003 | | *Tilapia cabrae*  Boulenger, 1899 | Mouth of the Bas Kouili River (R. Congo) | **Vagina non-sclerotized** | “Poorly developed heel, He = 3 ± (2-4)” ♠ | | [10]; p. 196, 197 |
| 1. *Cichlidogyrus rognoni*   Pariselle, Bilong Bilong & Euzet, 2003 | | *Oreochromis niloticus*  (Linnaeus, 1758) | Senegal River (Senegal) | **Vagina non-sclerotized** | “Developed heel, He = 5 (4-7)” ♠ | | [19]; p. 203, 204 |
| 1. *Cichlidogyrus sanjeani*   Pariselle & Euzet, 1997 | | *Sarotherodon occidentalis*  (Daget, 1962) | La Ramié, Bourouma River (Guinea) | **Vagina non-sclerotized** | “Heel, He = 3 (2-4)” ♠ | | [12]; p. 227, 229 |
| 1. *Cichlidogyrus sanseoi*   Pariselle & Euzet, 2004 | | *Hemichromis fasciatus*  Peters, 1857 | Kounougou River  (Ivory Coast) | “Vagina in four portions, from the aperture: simple tube followed by spiral (double pitch), then another simple straight portion ending in a second spiral (simple pitch), it was felt that no viable measurements could be done on this very complex vagina” ♥ | **No heel** | | [29]; p. 361, 362 |
| 1. *Cichlidogyrus sclerosus*   Paperna & Thurston, 1969 | | *Oreochromis mossambicus*  (Peters, 1852) | Kajansi (Uganda) | “Vagina hardly visible, slightly sclerotized, assuming shape of long, distally-widened tube” ♥ | “Basic portion of irregular shape” ♣ | | [23]; p. 23, 24; [1]; p. 166 |
| 1. *Cichlidogyrus sigmocirrus*   Pariselle, Bitja Nyom & Bilong Bilong, 2014 | | *Tylochromis sudanensis*  Daget, 1954 | Mamfé, Cross River (Cameroon) | **Vagina non-sclerotized** | “Penis starting in a bulb with reduced heel, He = 2 (2-3)” ♠ | | [13]; p. 261 |
| 1. *Cichlidogyrus slembroucki*   Pariselle & Euzet, 1998 | | *Tilapia buttikoferi*  (Hubrecht, 1881) | Kandiafara, Kogon River (Guinea) | “Thin vagina: Vg = 19 k 2.8 (15- 23) long and 3 k 0.5 (2-4) in diameter” ♠ | “Heel being very thin, He = 1 ± 0.1 (1-2)” ♠ | | [4]; p. 280, 281 |
| 1. *Cichlidogyrus testificatus*   Dossou, 1982 | | *Tilapia mariae*  Boulenger, 1899 | Ouémé (Benin) | “Le vagin filiforme et sinueux mesure 50-55 µm de longueur. Il forme souvent une petite boucle au tiers de sa longueur”; “Filiform and sinuous vagina of 50-55 µm long. It often forms a loop at its third portion” ♦♠ | “La pièce accessoire est rattachée à l’ampoule basale par un raccord subterminal de 10 µm”; “Sclerotization part of the accessory piece of 10 µm joins the basal bulb” ♦♣ | | [1]; p. 320, 321 |
| 1. *Cichlidogyrus teugelsi*   Pariselle & Euzet, 2004 | | *Hemichromis fasciatus*  Peters, 1857 | Kounougou River  (Ivory Coast) | “The vagina is a narrow and sinuous tube, with a slightly sclerotized plate close to the aperture: V = 72 ± 7.2 (61-88), v = 3 ± 0.3 (4-3)” ♠ | “Irregular heel” ♥ | | [29]; p. 362, 363 |
| 1. *Cichlidogyrus thurstonae*   Ergens, 1981 | | *Oreochromis niloticus*  (Linnaeus, 1758) | Nile River (Egypt) | “Vagina straight, irregularly shaped: V = 23 (20-25), v = 2 (1-2)” ♠ | “Blunt heel” ♣ | | [2]; p. 208, 209 |
| 1. *Cichlidogyrus tiberianus*   Paperna, 1960 | | *Coptodon zillii*  (Gervais, 1848) | Sea of Galilee (Israel) | “The vagina opens on the left side of the body near its center. The vaginal prop is represented by a plate and curled ducts both consisting of sclerotized unstainable substance” ♥ | “On one side, the rim of the funnel is serrated and a flat plate of variable shape is attached to it” ♥ | | [8]; p. 11, 12 |
| 1. *Cichlidogyrus tilapiae*   Paperna, 1960 | | *Sarotherodon galilaeus*  (Linnaeus, 1758) | Fish Culture Research Station at Dor (Israel) | **Vagina non-sclerotized** | **No heel**  “Without additional portion” | | [8]; p. 8, 9 |
| 1. *Cichlidogyrus vexus*   Pariselle & Euzet, 1995 | | *Coptodon guineensis*  (Günther, 1862) | Tiassalé, Bandama River (Ivory Coast) | “Vagina: V = 20.9 ± 1.2 (18-22), with thickened lining and short, narrow cylindric section” ♠ | “Accessory piece linked to base of penis by semicircular thickening” ♥ | | [3]; p. 196, 197 |
| 1. *Cichlidogyrus yanni*   Pariselle & Euzet, 1996 | | *Coptodon zillii*  (Gervais, 1848) | Kogon River (Guinea) | **Vagina non-sclerotized** | “Short thin heel” ♣ | | [6]; p. 119 |
| 1. *Cichlidogyrus zambezensis*   Douëllou, 1993 | | *Serranochromis macrocephalus*  (Boulenger, 1899) | Kariba Lake (Zimbabwe) | “Vagina orifice well sclerotized, triangular, hat-like (height: 5-8 µm, average 8; width of base: 7-11 µm, average 8); irregular part variable in size and shape but usually more or less round often present” ♠ | “Base with additional heavily sclerotized portion on which accessory piece is articulated; thin connection between this portion and accessory piece visible in some specimens” ♥ | | [18]; p. 179, 180 |
| **Tanganyikan *Cichlidogyrus* species** | | | | | |  |  |
| 1. *Cichlidogyrus aspiralis* n. sp.   Rahmouni, Vanhove & Šimková | | *Ophthalmotilapia nasuta*  (Poll & Matthes, 1962) | Magara (Burundi) | “Short sclerotized vagina consisting of a triangular and tubular section: V = 15.5 (15-16; n = 2); v = 6 (5-7; n = 2)” ♠ | “Long straight heel, He = 15.5 (14-18)” ♠ | | This paper |
| 1. Cichlidogyrus attenboroughi   Kmentová, Gelnar, Koblmüller & Vanhove, 2016 | | *Benthochromis horii*  Takahashi, 2008 | Bujumbura (Burundi) | **Vagina non-sclerotized** | **No heel** | | [30]; p. 5, 6 |
| 1. *Cichlidogyrus banyankimbonai*   Pariselle & Vanhove, 2015 | | *Simochromis diagramma*  (Günther, 1894) | Mukamba (D.R. Congo) | **Vagina non-sclerotized** | “Wide and short heel, He = 4.1 (2.1-5.7)” ♠ | | [31], p. 25 |
| 1. Cichlidogyrus brunnensis   Kmentová, Gelnar, Koblmüller & Vanhove, 2016 | | *Trematocara unimaculatum*  Boulenger, 1901 | Bujumbura (Burundi) | **Vagina non-sclerotized** | “Short heel, He = 7.6 (4.8-10) ♠ | | [30]; p. 3 |
| 1. *Cichlidogyrus buescheri*   Pariselle & Vanhove, 2015 | | *Interochromis loocki*  (Poll, 1949) | Kalambo Lodge (Zambia) | **Vagina non-sclerotized** | “Large heel of irregular shape, He = 8 (6-11)” ♠ | | [32]; p. 35, 37 |
| 1. Cichlidogyrus casuarinus   Pariselle, Muterezi Bukinga &Vanhove, 2015 | | *Bathybates minor*  Boulenger, 1906 | Mpala (D.R. Congo) | Large tubular vagina, with  proximal half surrounded by a thin sheath L=46  (36–59), l=7(5–8  “Large tubular vagina, with proximal half surrounded by a thin sheath: L = 46 (36–59), l = 7 (5–8)” ♠ | “Heel of the MA extremely long and slender; blunt distal end, often with constriction near to it, He = 47 (40-59)” ♠ | | [32] |
| 1. Cichlidogyrus *centesimus*   Vanhove, Volckaert & Pariselle, 2011 | | *Ophthalmotilapia ventralis*  (Boulenger, 1898) | Wonzye Point (Zambia) | No vagina in the original description but it has been reported later | “Straight and very long heel variable in length, He = 24 (11-47)” ♠ | | [33]; p. 258; [34] |
| 1. *Cichlidogyrus jeanloujustini* n. sp. Rahmouni, Vanhove & Šimková | | *Eretmodus marksmithi*  Burgess, 2012 | Mukuruka (Burundi) | **Vagina non-sclerotized** | “Reduced heel, He = 1-3 (2)” ♠ | | This paper |
| 1. *Cichlidogyrus discophonum* n. sp. Rahmouni, Vanhove & Šimková | | *Aulonocranus dewindti*  (Boulenger, 1899) | Nyaruhongoka (Burundi) | **Vagina non-sclerotized** | **No heel** ♠ | | This paper |
| 1. *Cichlidogyrus evikae* n. sp.   Rahmouni, Vanhove & Šimková | | *Tanganicodus irsacae*  Poll, 1950 | Mukuruka (Burundi) | **Vagina non-sclerotized** | “ Heel reduced to **inconspicuous** , He = 0-2 (1)” ♠ | | This paper |
| 1. *Cichlidogyrus frankwillemsi*   Pariselle & Vanhove, 2015 | | *Pseudosimochromis curvifrons* (Poll, 1942) | Kalambo Lodge (Zambia) | **Vagina non-sclerotized** | “Reduced to **no heel**, He = 0.9 (0.0-1.8)” ♠ | | [31]; p. 29 |
| 1. *Cichlidogyrus franswittei*   Pariselle & Vanhove, 2015 | | *Pseudosimochromis marginatus* (Poll, 1956) | Luhanga (D.R. Congo) | **Vagina non-sclerotized** | “Developed heel, He = 5.4 (3.1-9.1)” ♠ | | [31]; p. 28 |
| 1. *Cichlidogyrus georgesmertensi*   Pariselle & Vanhove, 2015 | | *Pseudosimochromis babaulti*  (Pellegrin, 1927) | Kyanza (D.R. Congo) | **Vagina non-sclerotized** | “Well-developed and club-shaped heel, He = 11.3 (7.1-15.8)” ♠ | | [31]; p. 27 |
| 1. *Cichlidogyrus gillardinae*   Muterezi Bukinga, Vanhove, Van Steenberge & Pariselle, 2012 | | *Astatotilapia burtoni*  (Günther, 1894) | Nyangara wetland, Uvira (D.R. Congo) | **Vagina non-sclerotized** | “Poorly developed heel, He = 5 (4-7)” ♠ | | [35]; p. 2052 |
| 1. *Cichlidogyrus gistelincki*   Gillardin, Vanhove, Pariselle, Huyse & Volckaert, 2012 | | “*Ctenochromis” horei*  (Günther, 1894) | Kalambo Lodge (Zambia) | **Vagina non-sclerotized** | “Trapezoid heel, He = 3.2 (2.9-3.5)” ♠ | | [36]; p. 310 |
| 1. *Cichlidogyrus glacicremoratus* n. sp. Rahmouni, Vanhove & Šimková | | *Ophthalmotilapia nasuta*  (Poll & Matthes, 1962) | Magara (Burundi) | **Vagina non-sclerotized** | “Irregular heel, He = 1-2 (1)” ♠ | | This paper |
| 1. *Cichlidogyrus irenae*   Gillardin, Vanhove, Pariselle, Huyse & Volckaert, 2012 | | *“Gnathochromis” pfefferi*  (Boulenger, 1898) | Kalambo Lodge (Zambia) | **Vagina non-sclerotized** | “Distinct blunt heel, He = 4.1 (3.6-4.4)” ♠ | | [36]; p. 308, 309 |
| 1. Cichlidogyrus *makasai*   Vanhove, Volckaert & Pariselle, 2011 | | *Ophthalmotilapia ventralis*  (Boulenger, 1898) | Wonzye Point (Zambia) | **Vagina non-sclerotized** | “Pronounced heel, He = 3 (2-4)” ♠ | | [33]; p. 256, 257 |
| 1. *Cichlidogyrus mbirizei*   Muterezi Bukinga, Vanhove, Van Steenberge & Pariselle, 2012 | | *Oreochromis tanganicae*  (Günther, 1894) | Kalemie fish market  (D.R. Congo) | “Very long, thin and spirally coiled vagina (double pitch); no valuable length measurement could be taken” ♥ | “Distinct heel, He = 10 (7-21)” ♠ | | [35]; p. 2053 |
| 1. *Cichlidogyrus milangelnari* n. sp.   Rahmouni, Vanhove & Šimková | | *Cyprichromis microlepidotus*  (Poll, 1956) | Nyaruhongoka (Burundi) | **Vagina non-sclerotized** | **No heel** | | This paper |
| 1. *Cichlidogyrus mulimbwai*   Muterezi Bukinga, Vanhove, Van Steenberge & Pariselle, 2012 | | *Tylochromis polylepis*  (Boulenger, 1900) | Mulembwe (D.R. Congo) | **Vagina non-sclerotized** | “Long heel, He = 9 (8-13)” ♠ | | [35]; p. 2054 |
| 1. *Cichlidogyrus muterezii*   Pariselle & Vanhove, 2015 | | *Simochromis diagramma*  (Günther, 1894) | Mugayo (D.R. Congo) | **Vagina non-sclerotized** | “Well-developed heel, He = 7 (5.1-8.4)” ♠ | | [31]; p. 23, 24 |
| 1. *Cichlidogyrus muzumanii*   Muterezi Bukinga, Vanhove, Van Steenberge & Pariselle, 2012 | | *Tylochromis polylepis*  (Boulenger, 1900) | Mulembwe (D.R. Congo) | **Vagina non-sclerotized** | “Not very prominent heel, He = 5 (4-7)” ♠ | | [35]; p. 2055 |
| 1. *Cichlidogyrus nshomboi*   Muterezi Bukinga, Vanhove, Van Steenberge & Pariselle, 2012 | | *Boulengerochromis microlepis*  (Boulenger, 1899) | Mulembwe (D.R. Congo) | No vagina in the original description but it has been reported later | “Very long heel (nearly as long as the penis), He = 22 (18-25)” ♠ | | [35]; p. 2056; [34] |
| 1. *Cichlidogyrus pseudoaspiralis*   n. sp. Rahmouni, Vanhove & Šimková | | *Aulonocranus dewindti*  (Boulenger, 1899) | Nyaruhongoka (Burundi) | **Vagina non-sclerotized** | “Long straight heel, He = 15.8 (14-17)” ♠ | | This paper |
| 1. *Cichlidogyrus raeymaekersi*   Pariselle & Vanhove, 2015 | | *Simochromis diagramma*  (Günther, 1894) | Mukamba (D.R. Congo) | **Vagina non-sclerotized** | “Short heel, He = 2.3 (1.3-4.0)” ♠ | | [31]; p. 22 |
| 1. *Cichlidogyrus rectangulus* n. sp.   Rahmouni, Vanhove & Šimková | | *Ophthalmotilapia nasuta*  (Poll & Matthes, 1962) | Magara (Burundi) | **Vagina non-sclerotized** | “Long and thick rectangular-shaped heel, He = 18-22 (20)” ♠ | | This paper |
| 1. *Cichlidogyrus schreyenbrichardorum*   Pariselle & Vanhove, 2015 | | *Interochromis loocki*  (Poll, 1949) | Kalambo Lodge (Zambia) | **Vagina non-sclerotized** | “Heel relatively small, slender but well-developed, He = 5 (4-6)” ♠ | | [32]; 35, 37 |
| 1. *Cichlidogyrus steenbergei*   Gillardin, Vanhove, Pariselle, Huyse & Volckaert, 2012 | | *Limnotilapia dardennii*  (Boulenger, 1899) | Kalambo Lodge (Zambia) | **Vagina non-sclerotized** | “Thin not very prominent heel, He = 5.4 (4.2-6.4)” ♠ | | [36]; p. 307, 309 |
| 1. *Cichlidogyrus sturmbaueri*   Vanhove, Volckaert & Pariselle, 2011 | | *Ophthalmotilapia ventralis*  (Boulenger, 1898) | Wonzye Point (Zambia) | **Vagina non-sclerotized** | “Short heel, He = 6 (4-7)” ♠ | | [33]; p. 259, 260 |
| 1. Cichlidogyrus vandekerkhovei   Vanhove, Volckaert & Pariselle, 2011 | | *Ophthalmotilapia ventralis*  (Boulenger, 1898) | Wonzye Point (Zambia) | **Vagina non-sclerotized** | “Well-developed heel, He = 5 (4-6)” ♠ | | [33]; p. 255, 256 |
| 1. *Cichlidogyrus vealli*   Pariselle & Vanhove, 2015 | | *Interochromis loocki*  (Poll, 1949) | Kalambo Lodge (Zambia) | **Vagina non-sclerotized** | “Short, stubby but well-developed heel, He = 5 (4-6)” ♠ | | [32]; p. 35, 38 |

**References**.

1. Dossou C, Birgi E. Monogènes parasites d’*Hemichromis fasciatus* Peters, 1857 (Teleostei, Cichlidae). Ann Sci Nat Zool. 1984;6:101–9.
2. Ergens R. Nine species of the genus *Cichlidogyrus* Paperna, 1960 (Monogenea: Ancyrocephalinae) from Egyptian fishes. Folia Parasitol. 1981;28:205–14.
3. Pariselle A, Euzet L. Gill parasites of the genus *Cichlidogyrus* Paperna, 1960 (Monogenea, Ancyrocephalidae) from *Tilapia guineensis* (Bleeker, 1862), with descriptions of six new species. Syst Parasitol. 1995;30:187–98.
4. Pariselle A, Euzet L. Five new species of *Cichlidogyrus* (Monogenea: Ancyrocephalidae) from *Tilapia brevimanus*, *T. buttikoferi* and *T. cessiana* from Guinea, Ivory Coast and Sierra Leone (West Africa). Folia Parasitol. 1998;45:275–82.
5. Birgi E, Euzet L. Monogènes parasites des poissons des eaux douces du Cameroun. Présence des genres *Cichlidogyrus* et *Dactylogyrus* chez *Aphyosemion* (Cyprinodontidae). Bull Soc Zool Fr. 1983;108:101–6.
6. Pariselle A, Euzet L. *Cichlidugyrus* Paperna, 1960 (Monogenea, Ancyrocephalidae): gill parasites from West African Cichlidae of the subgenus *Coptodon* Regan, 1920 (Pisces), with descriptions of six new species. Syst Parasitol. 1996;34:109–24.
7. Pariselle A, Euzet L. Trois Monogènes nouveaux parasites branchiaux de *Pelmatochromis buettikoferi* (Steindachner, 1895) (Cichlidae) en Guinée. Parasite. 1995;2:203–9.
8. Paperna I. Studies on monogenetic trematodes in Israel. 2 Monogenetic trematodes of cichlids. Bamidgeh, Bull Fish Cult Isr. 1960;12:20–33.
9. Pariselle A, Bitja Nyom AR, Bilong Bilong CF. Checklist of the ancyrocephalids (Monogenea) parasitizing *Tilapia* species in Cameroon, with the description of three new species. Zootaxa. 2013;3599:78–86.
10. Pariselle A, Euzet L. Four new species of *Cichlidogyrus* (Monogenea: Ancyrocephalidae), gill parasites of *Tilapia cabrae* (Teleostei: Cichlidae), with discussion on relative length of haptoral sclerites. Folia Parasitol. 2003;50:195–201.
11. Pariselle A, Euzet L. Three new species of *Cichlidogyrus* Paperna, 1960 (Monogenea: Ancyrocephalidae) parasitic on *Tylochromis jentinki* (Steindachner, 1895) (Pisces, Cichlidae) in West Africa. Syst Parasitol. 1994;29:229–34.
12. Pariselle A, Euzet L. New species of *Cichlidogyrus* Paperna, 1960 (Monogenea, Ancyrocephalidae) from the gills of *Sarotherodon occidentalis* (Daget) (Osteichthyes, Cichlidae) in Guinea and Sierra Leone (West Africa). Syst Parasitol. 1997;38:221–30.
13. Pariselle A, Bitja Nyom AR, Bilong Bilong CF. Four new species of *Cichlidogyrus* (Monogenea, Ancyrocephalidae) from *Sarotherodon mvogoi* and *Tylochromis sudanensis* (Teleostei, Cichlidae) in Cameroon. Zootaxa. 2014;3881:258–66.
14. Paperna I. Parasitic helminths of inland-water fishes in Israel. Isr J Zool. 1964;13:1–26.
15. Jorissen MWP, Pariselle A, Huyse T, Vreven EJ, Snoeks J, Volckaert FAM, Manda AC, Kasembele GK, Artois T, Vanhove MPM. Diversity and host specificity of monogenean gill parasites (Platyhelminthes) of cichlid fishes in the Bangweulu-Mweru ecoregion. J. Helminthol.; 2017;1–21.
16. Dossou C, Birgi E. Monogènes parasites d’*Hemichromis fasciatus* Peters, 1857 (Teleostei, Cichlidae). Ann Sci Nat Zool. 1984;6:101–9.
17. Paperna I. Monogenetic trematodes collected from fresh water fish in Ghana. Second report. Bamidgeh, Bull Fish Cult Isr. 1968;20:80–100.
18. Douëllou L. Monogeneans of the genus *Cichlidogyrus* Paperna, 1960 (Dactylogyridae: Ancyrocephalinae) from cichlid fishes of Lake Kariba (Zimbabwe) with descriptions of five new species. Syst Parasitol. 1993;25:159–86.
19. Pariselle A, Bilong Bilong CF, Euzet L. Four new species of *Cichlidogyrus* Paperna, 1960 (Monogenea, Ancyrocephalidae), all gill parasites from African mouthbreeder tilapias of the genera *Sarotherodon* and *Oreochromis* (Pisces, Cichlidae), with a redescription of *C. thurstonae* Ergens, 1981. Syst Parasitol. 2003;56:201–10.
20. Řehulková E, Mendlová M, Šimková A. Two new species of *Cichlidogyrus* (Monogenea: Dactylogyridae) parasitizing the gills of African cichlid fishes (Perciformes) from Senegal: morphometric and molecular characterization. Parasitol Res. 2013;112:1399–1410.
21. Paperna I, Thurston JP. Monogenetic Trematodes collected from cichlid fish in Uganda; including the description of five new species of *Cichlidogyrus*. Rev Zool Bot Afr. 1969;79:15–33.
22. Price CE, Kirk RG. First description of a monogenetic trematode from Malawi. Rev Zool Bot Afr. 1967;16:137–44.
23. Paperna I, Thurston JP. Monogenetic Trematodes collected from cichlid fish in Uganda; including the description of five new species of *Cichlidogyrus*. Rev Zool Bot Afr. 1969;79:15–33.
24. Birgi E, Lambert A. À propos de *Cichlidogyrus euzeti* Birgi & Lambert, 1986 décrit au Cameroon chez un Nandidae *Polycentropsis abbreviata* Boulenger, 1901. Ann Parasitol Hum Comp. 1987;62:103.
25. N’Douba V, Thys van den Audenaerde DFE, Pariselle A. Description d’une espèce nouvelle de Monogène ectoparasite branchial de *Tilapia guineensis* (Bleeker, 1862) (Cichlidae) en Côte d’Ivoire. J Afr Zool. 1997;111:429–33.
26. Paperna I. Monogenetic trematodes collected from fresh water fish in southern Ghana. Bamidgeh, Bull Fish Cult Isr. 1965;17:107–15.
27. Birgi E, Lambert, A. Présence chez un Nandidae (Téléostéen), *Polycentropsis abbreviata* Boulenger, 1901, du genre *Cichlidogyrus* (Monogenea, Monopisthocotylea, Ancyrocephalidae). Description de *Cichlidogyrus nandidae* n. sp. Ann Parasitol Hum Comp. 1986;61:521–28.
28. Price CE, Peebles HE, Bamford T. The monogenean parasites of African fishes. IV. Two new species from South African hosts. Rev Zool Bot Afr. 1969;79:117–24.
29. Pariselle A, Euzet L. Two new species of *Cichlidogyrus* Paperna, 1960 (Monogenea, Ancyrocephalidae) gill parasites on *Hemichromis* *fasciatus* (Pisces, Cichlidae) in Africa, with remarks on parasite geographical distribution. Parasite. 2004;11:359–64.
30. Kmentová N, Gelnar M, Koblmüller S, Vanhove MPM. Deep-water parasite diversity in Lake Tanganyika: description of two new monogenean species from benthopelagic cichlid fishes. Parasit Vectors. 2016;9:426.
31. Van Steenberge M, Pariselle A, Huyse T, Volckaert FAM, Snoeks J, Vanhove MPM. Morphology, molecules, and monogenean parasites: an example of an integrative approach to cichlid biodiversity. PLoS One. 2015;10:e0124474.
32. Pariselle A, Muterezi Bukinga F, Van Steenberge M, Vanhove MPM. Ancyrocephalidae (Monogenea) of Lake Tanganyika. IV. *Cichlidogyrus* parasitizing species of Bathybatini (Teleostei, Cichlidae): reduced host-specificity in the deepwater realm? Hydrobiologia. 2015;748:99–119.
33. Vanhove MPM, Volckaert FAM, Pariselle A. Ancyrocephalidae (Monogenea) of Lake Tanganyika: I: Four new species of *Cichlidogyrus* from *Ophthalmotilapia ventralis* (Teleostei: Cichlidae), the first record of this parasite family in the basin. Zool. (Curitiba, Impresso). 2011;28:253–63.
34. Pariselle A, Muterezi Bukinga F, Van Steenberge M, Vanhove MPM. Ancyrocephalidae (Monogenea) of Lake Tanganyika. IV. *Cichlidogyrus* parasitizing species of Bathybatini (Teleostei, Cichlidae): reduced host-specificity in the deepwater realm? Hydrobiologia. 2015;748:99–119.
35. Muterezi Bukinga F, Vanhove MPM, Van Steenberge M, Pariselle A. Ancyrocephalidae (Monogenea) of Lake Tanganyika: III: *Cichlidogyrus* infecting the world’s biggest cichlid and the non-endemic tribes Haplochromini, Oreochromini and Tylochromini (Teleostei, Cichlidae). Parasitol Res. 2012;111:2049–61.
36. Gillardin C, Vanhove MPM, Pariselle A, Huyse T, Volckaert FAM. Ancyrocephalidae (Monogenea) of Lake Tanganyika: II: description of the first *Cichlidogyrus* spp. parasites from Tropheini fish hosts (Teleostei, Cichlidae). Parasitol Res. 2012;110:305–13.
